# A GATA Transcription Factor Recruits Hda1 in Response to Reduced Tor1 Signaling to Establish a Hyphal Chromatin State in *Candida albicans*

**DOI:** 10.1371/journal.ppat.1002663

**Published:** 2012-04-19

**Authors:** Yang Lu, Chang Su, Haoping Liu

**Affiliations:** Department of Biological Chemistry, University of California, Irvine, California, United States of America; Carnegie Mellon University, United States of America

## Abstract

*Candida albicans* is an important opportunistic fungal pathogen of immunocompromised individuals. One critical virulence attribute is its morphogenetic plasticity. Hyphal development requires two temporally linked changes in promoter chromatin, which is sequentially regulated by temporarily clearing the transcription inhibitor Nrg1 upon activation of the cAMP/PKA pathway and promoter recruitment of the histone deacetylase Hda1 under reduced Tor1 signaling. Molecular mechanisms for the temporal connection and the link to Tor1 signaling are not clear. Here, through a forward genetic screen, we report the identification of the GATA family transcription factor Brg1 as the factor that recruits Hda1 to promoters of hypha-specific genes during hyphal elongation. *BRG1* expression requires both the removal of Nrg1 and a sub-growth inhibitory level of rapamycin; therefore, it is a sensitive readout of Tor1 signaling. Interestingly, promoters of hypha-specific genes are not accessible to Brg1 in yeast cells. Furthermore, ectopic expression of Brg1 cannot induce hyphae, but can sustain hyphal development. Nucleosome mapping of a hypha-specific promoter shows that Nrg1 binding sites are in nucleosome free regions in yeast cells, whereas Brg1 binding sites are occupied by nucleosomes. Nucleosome disassembly during hyphal initiation exposes the binding sites for both regulators. During hyphal elongation, Brg1-mediated Hda1 recruitment causes nucleosome repositioning and occlusion of Nrg1 binding sites. We suggest that nucleosome repositioning is the underlying mechanism for the yeast-hyphal transition. The hypha-specific regulator *Ume6* is a key downstream target of Brg1 and functions after Brg1 as a built-in positive feedback regulator of the hyphal transcriptional program to sustain hyphal development. With the levels of Nrg1 and Brg1 dynamically and sensitively controlled by the two major cellular growth pathways, temporal changes in nucleosome positioning during the yeast-to-hypha transition provide a mechanism for signal integration and cell fate specification. This mechanism is likely used broadly in development.

## Author Summary

*Candida* is part of the gut microflora in healthy individuals, but can disseminate and cause systemic disease when the host's immune system is suppressed. Its ability to grow as yeast and hyphae in response to environmental cues is a major virulence attribute. Hyphal development requires temporary clearing of the transcription inhibitor Nrg1 upon activation of cAMP/PKA for initiation and promoter recruitment of the histone deacetylase Hda1 under reduced Tor1 signaling for maintenance. Here, we show that, during hyphal initiation when Nrg1 is gone, expression of the GATA family transcription factor Brg1 is activated under reduced Tor1 signaling. Accumulated Brg1 recruits Hda1 to hyphal promoters to reposition nucleosomes, leading to obstruction of Nrg1 binding sites and sustained hyphal development. The nucleosome repositioning during the yeast-hyphal transition provides a mechanism for temporal integration of extracellular signals and cell-fate specification. The hypha-specific transcription factor Ume6 functions after Brg1 in this succession of feed-forward regulation of hyphal development. Since misregulation of either Nrg1 or Ume6 causes altered virulence, and Brg1 regulates both Nrg1 accessibility and Ume6 transcription, our findings should provide a better understanding of how *Candida* controls its morphological program in different host niches to exist as a commensal and a pathogen.

## Introduction

*Candida albicans* is a major opportunistic fungal pathogen of humans [1], [2]. In most healthy individuals *C. albicans* exists as a harmless commensal in the oral cavity and the gastrointestinal and urogenital tracts. But the fungus can cause mucosal infections and systemic disease in immunocompromised people. One critical virulence attribute of *C. albicans* is its ability to undergo hyphal development in response to environmental cues. Mutants that are defective in hyphal formation display much reduced virulence in animal models of systemic candidiasis [3], [4]. Hyphal morphogenesis is coupled with virulence as genes that control hyphal morphology are co-regulated with genes encoding virulence factors such as proteases and adhesins [4]. For example, *HWP1*, *ALS3*, and *RBT5* encode cell wall proteins that are important for adhesion to host cells and iron acquisition from the host [5]–[8]. The transcription factor Ume6, specifically expressed during hyphal development, controls the level and duration of hypha-specific genes and is important for hyphal elongation [9]–[11]. Hyphal morphogenesis and cell chain formation are under the control of another hypha-specific gene that encodes the G_1_ cyclin-related protein Hgc1 [12]–[16].

Hyphal development is regulated by multiple signal transduction pathways. Among them, the cAMP-dependent protein kinase A (PKA) pathway is essential for morphogenesis and virulence [17]. We recently showed that activation of the hyphal transcriptional program involves two phases of temporal dynamic changes in promoter chromatin [18]. Initiation requires a rapid but temporary disappearance of Nrg1, a major repressor of hyphal morphogenesis [19], [20], via activation of the cAMP-PKA pathway [18]. Nrg1 disappearance from the promoters of hypha-specific genes is correlated with dissociation of the Rpd3 histone deacetylase from the promoters, increase in H4 acetylation, nucleosome disassembly, and transcriptional activation. Maintenance requires the recruitment of the Hda1 histone deacetylase to promoters under reduced Tor1 (target of rapamycin) signalling [18]. Hda1 deacetylates a subunit of the NuA4 histone acetyltransferase module, leading to eviction of the NuA4 acetyltransferase module and chromatin remodelling that blocks Nrg1 access to the promoters of hypha-specific genes. Promoter recruitment of Hda1 for hyphal maintenance happens only during the period when Nrg1 is gone. Such temporally linked regulation of promoter chromatin provides a unique mechanism for integrating multiple signals in the regulation of gene expression and phenotypic plasticity during hyphal development.

*C. albicans* contains a conserved Tor1 protein kinase [21], [22]. Gln3 and Gat1, two GATA family transcription activators for genes that are subject to nitrogen catabolite repression, are targets of the TOR kinases in *Saccharomyces cerevisiae* [23]. Their homologs in *C. albicans* [24], [25], however, are not important for hyphal maintenance. The transcription factor that recruits Hda1 to the promoters of hypha-specific genes has not been identified. Furthermore, the molecular mechanism for the temporal connection between hyphal initiation and elongation is not clear. Here, we report, through a forward genetic screen the identification of the GATA family transcription factor Gat2, recently found as a biofilm regulator Brg1 [26], as the one that recruits Hda1 to promoters of hypha-specific genes. *BRG1* expression is a sensitive readout of Tor1 signalling level in *C. albicans*, and Brg1 expression during hyphal elongation leads to nucleosome repositioning. Different nucleosome positions at the promoters of hypha-specific genes lead to different accessibility of Nrg1 and Brg1 to their DNA-binding sites at the promoters. This provides molecular insights to the sequential and integrative nature of promoter chromatin regulation. We further show that Brg1 functions before Ume6 in a feed-forward loop that regulates the yeast-to-hypha transition in *C. albicans*.

## Results

### Brg1 is required for promoter recruitment of Hda1 under reduced Tor1 signaling during hyphal elongation

**Figure 1:**
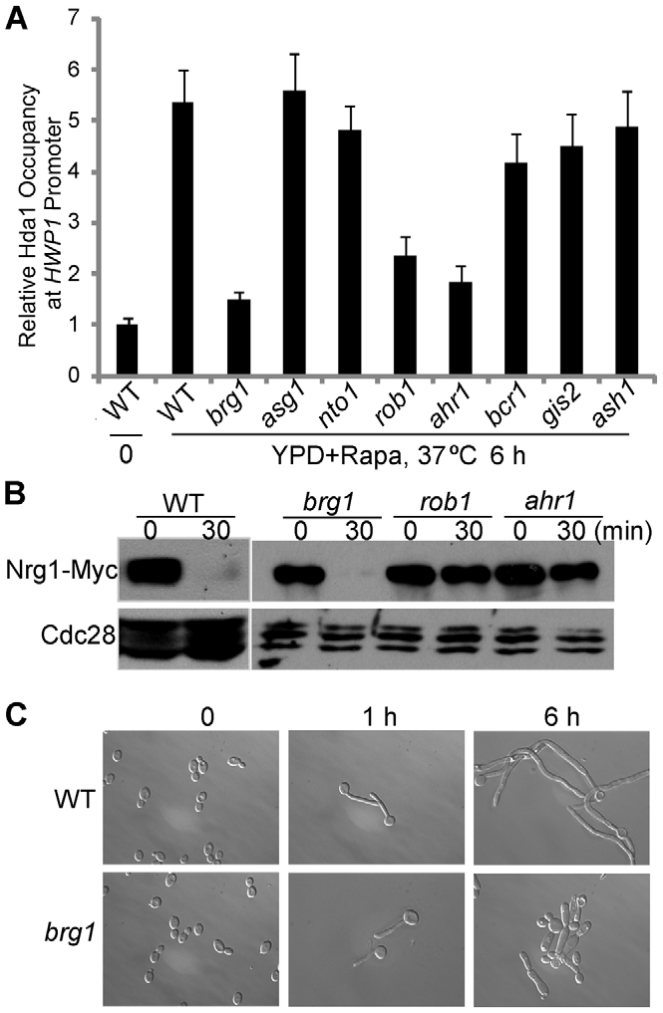
*brg1* is the only mutant found to be defective in Hda1 promoter recruitment. (A). ChIP of Hda1-Myc in mutants defective in hyphal elongation. Wild type and indicated mutant cells carrying Hda1-Myc were diluted into YPD+10 nM rapamycin medium at 37°C for 6 h. ChIP DNA was quantitated as described [18] by qPCR with primers at the UAS region of *HWP1* [30]. The 0 h values of wild type cells were set to be 1.00. The ChIP data show the average of three independent qPCR experiments with error bars representing the SEM. (B) Western analysis of Nrg1-Myc. *brg1*, *rob1*, and *ahr1* mutant cells carrying Nrg1-Myc were diluted into YPD medium at 37°C for 30 min. (C). Overnight cultures of wild type (SN250) and *brg1* mutant were diluted 1∶250 fold into YPD medium at 37°C. 5 nM rapamycin was added after 1 hour to YPD medium. Cells were collected at 0 h, 1 h, and 6 h for cell morphology analysis.

To uncover how rapamycin regulates hyphal elongation, we used a forward genetic screen to identify transcription factors responsible for recruitment of Hda1 under reduced Tor1 signaling. Based on the phenotype of the *hda1* mutant, we expected the mutants of interest to be capable of germ-tube formation, but defective in sustained hyphal growth. Furthermore, they were expected to block rapamycin-mediated hyphal elongation. Mutants defective in hyphal elongation, but with defects which could be suppressed by rapamycin, were not studied further as their gene products were expected to act upstream or parallel to Tor1 signaling. We screened a knockout library of 165 transcription factor genes in *C. albicans* [27] for mutants defective in hyphal elongation in the presence of rapamycin. Only 8 mutants showed defects to varying extents in hyphal elongation in the presence of rapamycin, and could form germ tubes during hyphal initiation (Table 1). Among the 8 mutants, we found that promoter recruitment of Hda1 in the presence of rapamycin was dramatically reduced in *brg1*, *rob1*, and *ahr1* mutants compared to the wild-type strain by chromatin immunoprecipitation (ChIP) (Figure 1A), and Hda1 protein levels were not reduced in those mutants (data not shown). Because promoter recruitment of Hda1 could only be established within the time window of reduced Nrg1 [18], any defects in Nrg1 down-regulation would also block Hda1 recruitment. To exclude this possibility, a Western of Nrg1-Myc was performed in these three mutants. Nrg1 protein level decreased sharply in the *brg1* mutant at 30 min upon hyphal induction as in the wild-type strain. But no obvious change in Nrg1 level was observed in the *rob1* or *ahr1* mutant (Figure 1B). This is consistent with the efficient germ tube formation (∼90%) observed in *brg1* cells (Figure 1C). The *brg1* mutant showed a similar defect in hyphal elongation and Hda1 promoter recruitment in response to serum (Figure S1). The Brg1-dependent Hda1 recruitment to the UAS region of *HWP1* is shown in Figure 1A, and to the UAS regions of *ALS3* and *ECE1* shown in Figure S2. Therefore, Brg1 is the only transcription factor specifically required for the recruitment of Hda1 to hypha-specific promoters, but not involved in Nrg1 down-regulation during hyphal initiation. Previous genome-wide mutant screens have identified Brg1 as a general positive regulator of morphogenesis [27] and a *brg1* mutant carrying a transposon insertion proximal to the ORF was found to be hyperfilamentous [28]. Our results provide a molecular function of Brg1 in filamentous growth.

**Table 1:** Transcription factor mutants defective in hyphal maintenance in rapamycin.

Gene name	Description*	Germ tube formation in YPD	Hyphal elongation in YPD+rapamycin
Wild type		+ + + + +	+ + + + +
*NTO1*	Subunit of the NuA3 histone acetyltransferase complex that acetylates histone H3; contains PHD finger domain that interacts with methylated histone H3	+ + + + +	+ + +
*GIS2*	Putative transcription factor; expression is increased in high iron and reduced upon yeast-hyphal switch; null mutant exhibits sensitivity to sorbitol, 5-fluorocytosine, and cold temperatures	+ + + + +	+ + +
*ASH1*	GATA-like transcription factor; localizes to nuclei of daughter cells and hyphal tip cells; mRNA localization is mediated by binding to She3p; required for wild-type virulence and filamentous growth	+ + + + +	+ + +
*BRG1*	Putative DNA-binding transcription factor; similar to *S. cerevisiae* Gat2p; transposon mutation affects filamentous growth	+ + + + +	+
*ASG1*	Gal4p family zinc-finger transcription factor with similarity to *S. cerevisiae* Asg1p	+ + + + +	+ +
*ROB1*	Putative protein of unknown function; null mutant displays abnormal colony morphology and invasive growth; caspofungin repressed	+ + +	+
*AHR1*	Zinc-finger transcription factor involved in regulation of adhesion genes; involved in white-opaque switch; forms complex with Mcm1p; null mutant displays sensitivity to 5-fluorocytosine and to lithium chloride	+ +	+
*BCR1*	C2H2 zinc finger transcription factor required for wild-type biofilm formation; mutation affects filamentous growth; regulates cell-surface-associated genes; filament induced; Tup1p-, Tec1p-, Mnl1p-regulated; mRNA binds to She3p	+ + +	+ +

*: All descriptions are from the *Candida* Genome Database. + indicates levels of filamentation.

C. *albicans* Brg1 is most homologous in protein sequence to *S. cerevisiae* Gat2 by BLAST analysis. Both proteins contain a GATA family zinc finger motif most similar to Gat3, Gat4, Gat1 and Gln3 of *S. cerevisiae*. Functions and regulations of Gat2, Gat3, and Gat4 in *S. cerevisiae* are not well studied. Gln3 and Gat1 are phosphorylated by the TOR kinases [23], and sequestered in the cytoplasm. In response to rapamycin treatment, Gln3 and Gat1 increasingly accumulate in the nucleus, where they bind to DNA and activate transcription of nitrogen catabolite repression-sensitive genes [29]. To examine if Brg1 accumulates in the nucleus in response to reduced Tor1 signaling, we localized Brg1 by indirect immunofluorescence. Brg1 was tagged at its N terminus with 13 Myc, and its expression was under the control of the *MAL2* promoter. Unlike Gln3 and Gat1, Myc-Brg1 displayed nuclear localization under all conditions with or without rapamycin (Figure S3).

### Brg1 interacts with Hda1 and binds to the promoters of hypha-specific genes in a rapamycin-dependent manner

**Figure 2:**
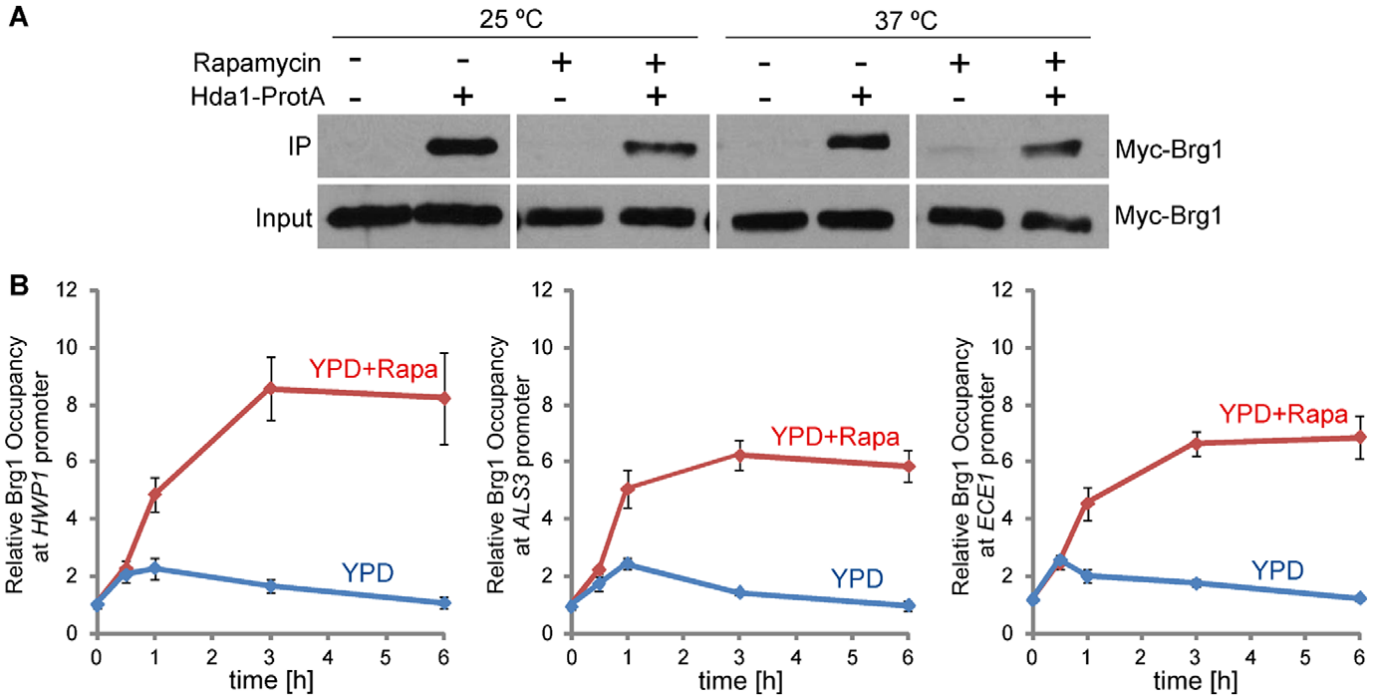
Brg1 interacts with Hda1 *in vivo* and binds to promoters of hypha-specific genes. (A). Cells of wild type transformed with Myc-Brg1 and Hda1-Protein A (HLY4081) or with only Myc-Brg1 (HLY3636) were grown in YEPMaltose medium at 25°C or 37°C in the presence or absence of 10 nM rapamycin. Protein lysates were subjected to immunoprecipitation with IgG beads (Sigma). The precipitated proteins (IP) and cell lysates (input) were analyzed by Western blotting. (B). Brg1 is present at the *HWP1* promoter in a rapamycin-containing medium. Cells of wild-type strain carrying Brg1-Myc (HLY4082) were diluted into YPD medium at 37°C in the presence or absence of 10 nM rapamycin. ChIP DNA was quantitated by qPCR as described in Figure 1. The ChIP data show the average of three independent qPCR experiments with error bars representing the SEM.

To determine if Brg1 interacts with Hda1, Hda1 was fused at its C-terminus with a TAP tag of two copies of protein A sequence followed by calmodulin binding protein [30]. Using strains carrying the Myc-Brg1 under the *MAL2* promoter, we found that immunoprecipitation of Hda1 with IgG beads was able to pull down Myc-Brg1 (Figure 2A). The interaction was not regulated by growth forms, and was independent of rapamycin and temperature (Figure 2A).

Since Brg1 is the transcription factor required for the promoter recruitment of Hda1 and it interacts with Hda1 *in vivo*, we anticipated that Brg1 directly binds to the promoters of hypha-specific genes. We tagged Brg1 at the C-terminus with 13 Myc under its endogenous promoter for Brg1 ChIP experiments and performed a time course ChIP during hyphal induction. Brg1-myc was not detected at the promoters of hypha-specific genes in yeast cells from the starting culture. During hyphal growth at 37°C, Brg1-myc became associated with the UAS regions of the hypha-specific promoters in a rapamycin-dependent manner (Figure 2B), very similar to rapamycin-dependent promoter recruitment of Hda1 [18]. Our results suggest that Brg1 recruits Hda1 to hyphal gene promoters to sustain hyphal development. This Tor1-mediated regulation could be at the level of Brg1 expression, stability, and/or promoter binding.

### Removal of Nrg1 inhibition and rapamycin are both required for *BRG1* expression

**Figure 3:**
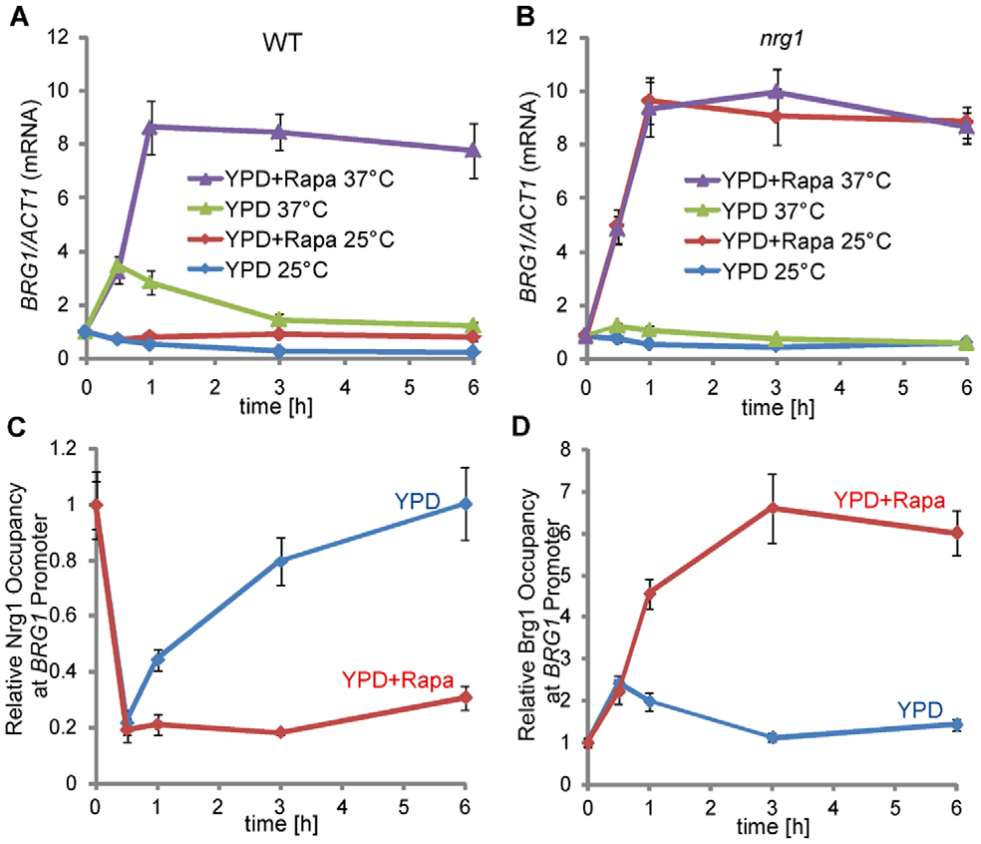
*BRG1* expression is controlled by both removal of Nrg1 repression and rapamycin-dependent activation. qRT-PCR analysis of *BRG1* expression in wild type (A) and in the *nrg1* mutant (B). Cells were diluted 1∶250 fold into pre-warmed YPD medium at 25°C or 37°C in the presence or absence of 10 nM rapamycin. *BRG1* mRNA levels were determined by qRT-PCR. The signals obtained from *ACT1* mRNA were used for normalization. The 0 h normalized value of *BRG1/ACT1* for the wild type was set to be 1.00, and used for normalization of all other values in A and B. ChIP time courses of Nrg1-Myc (HLY3922) and Brg1-Myc (HLY4082) are shown in C and D, respectively. Cells were diluted into YEPD medium at 37°C in the presence or absence of 10 nM rapamycin. ChIP DNA were quantitated by qPCR with primers at −1959∼−1710 bp of the *BRG1* promoter as described in Figure 1. All data show an average of three independent qRT-PCR or qPCR experiments with error bars representing the SEM.

We next examined whether Brg1 protein levels changed in response to rapamycin. The protein level of Brg1-myc under its endogenous promoter was low, but detectable in cells from overnight cultures. It increased rapidly at 1 h upon hyphal induction at 37°C and stayed at a high level in rapamycin-containing medium during hyphal elongation, but returned to a low level in the absence of rapamycin (Figure S4A), suggesting that reduced Tor1 signaling was required for a sustained high level of Brg1 expression. However, rapamycin alone was not sufficient for a high level of Brg1, as Brg1 levels were low at 25°C even in the presence of rapamycin (Figure S4A). Brg1 protein was unstable, but its stability was not regulated by temperature or rapamycin (Figure S3B). This suggested that rapamycin-dependent accumulation of Brg1 protein likely reflected the transcriptional regulation of *BRG1* by the Tor1 pathway. Similar to its protein levels, we found that *BRG1* expression was activated by a shift in temperature to 37°C and maintained at a high level in the presence of rapamycin (Figure 3A). In the absence of rapamycin, *BRG1* expression level increased briefly when cells were inoculated to YPD at 37°C, but decreased afterwards (Figure 3A). *BRG1* expression level remained low at 25°C regardless of the presence of rapamycin. Since Nrg1 is temporarily cleared when cells are inoculated into fresh medium at 37°C [18], a prerequisite condition for the induction in *BRG1* expression, we examined whether the down-regulation of Nrg1 is essential for the activation of *BRG1* expression. We found that ectopic expression of *NRG1* under the *MAL2* promoter completely blocked the induction of *BRG1* in YPD medium with rapamycin at 37°C (Figure S5). Furthermore, rapamycin could induce *BRG1* expression independent of temperature in an *nrg1* mutant (Figure 3B). These results suggest that Nrg1 removal is required for rapamycin-induced *BRG1* expression during hyphal elongation. Unlike hyphal genes, *BRG1* transcript or Brg1 protein is present in the starting yeast cell, although at a low level. Furthermore, removing Nrg1 inhibition is not sufficient for the induction of *BRG1* expression, as *BRG1* transcript levels at time 0 were similar between the *nrg1* mutant and the wild-type strain (Figure 3B vs. 3A. the value at time 0 in A was set to 1 and used for normalization in both A and B), and *BRG1* cannot be induced in the *nrg1* mutant in the absence of rapamycin (Figure 3B). Therefore, relief of Nrg1 inhibition by the activation of the cAMP/PKA pathway and reduced Tor1 signaling are both required for *BRG1* expression; neither one is sufficient.

Similar to hypha-specific genes, we predicted that Nrg1 and Brg1 can bind to the *BRG1* promoter, and Brg1 recruitment of Hda1 can remodel the promoter chromatin to block Nrg1 from binding onto the *BRG1* promoter during hyphal elongation. Nrg1 has been reported to bind to [A/C][A/C/G]CCCT, CCCCT or CCCT/C containing sequences [20] and Brg1 binds to the DNA sequence [A/C]GGTA[C/A] [26]. We analyzed the intergenic region upstream of *BRG1* (∼10 kb) and found 3 regions of ∼250 bp long that contain binding motifs for both Nrg1 and Brg1 (Figure S6). The ChIP DNA of Nrg1-Myc at time 0 (yeast cells) and Brg1-Myc at 6 h in the presence of rapamycin at 37°C (hyphal cells) were analyzed by qPCR at these regions (Figure S6). Among them, the region −1959∼−1710 bp showed much higher ChIP signals for both Nrg1-Myc and Brg1-Myc than the other regions (Figure S6). This region was therefore considered as a potential UAS region for *BRG1*, and was used for subsequent ChIP time course experiments. As expected, Nrg1 dissociated rapidly from the *BRG1* promoter during hyphal initiation and remained unbound in a rapamycin-dependent manner; Brg1 bound to the promoter in the presence of rapamycin only during hyphal elongation (Figure 3CD), similar to its temporal association with the promoters of hypha-specific genes.

### Ectopically expressed Brg1 can bind to the promoters of many hypha-specific genes independent of rapamycin, while binding to its own promoter is rapamycin-dependent

**Figure 4:**
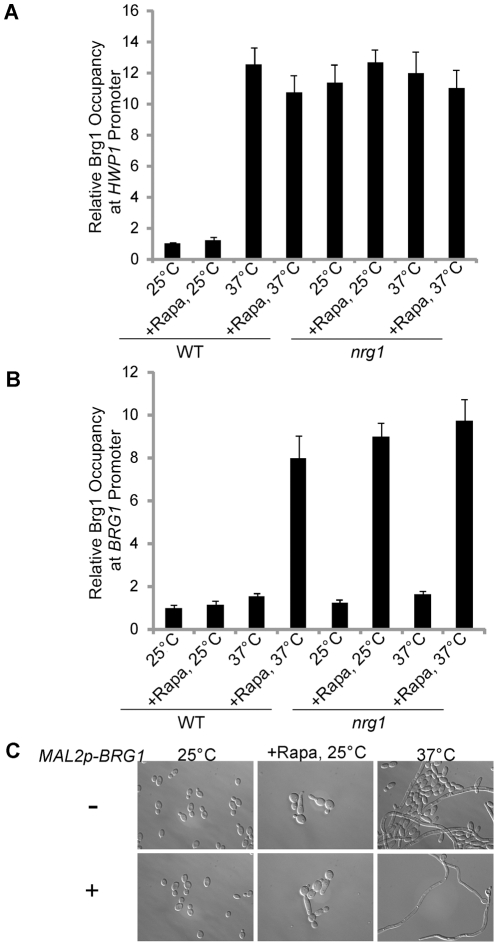
Ectopically expressed Brg1 sustains hyphal transcription independent of rapamycin, but cannot bind the *HWP1* or *BRG1* promoters in yeast cells. Ectopically expressed Brg1 could not bind to the *HWP1* promoter (A) or the *BRG1* promoter (B) when Nrg1 was present. Cells of wild type (HLY3636) and *nrg1* (HLY4080) carrying Brg1-Myc under the *MAL2* promoter were diluted into YEPMaltose medium at 25°C or 37°C in the presence or absence of 10 nM rapamycin for 6 h. ChIP DNA was quantitated by qPCR as described in Figure 3. All data show the average of three independent qRT-PCR or qPCR experiments with error bars representing the SEM. (C). Cells of wild type carrying Myc-Brg1 under the *MAL2* promoter were diluted 1∶250 fold into YEPMaltose medium at 25°C or 37°C for 6 h in the presence or absence of 10 nM rapamycin.

In strains carrying Brg1-myc under its endogenous promoter, we found that Brg1 binding to the promoters of hypha-specific genes and *BRG1* is rapamycin-dependent (Figures 2B and 3D). However, *BRG1* expression also required rapamycin (Figure 3AB), which could lead to the rapamycin-dependent promoter binding observed with Brg1-myc under its own promoter (Figures 2B & 3D). Since *BRG1* expression also requires the initial removal of Nrg1 and subsequent binding of Brg1 (Figure 3B) similar to hypha-specific promoters, we wanted to examine whether Brg1 DNA binding is under the control of Tor1. Ectopically expressed Brg1-myc under the *MAL2* promoter was used in ChIP experiments to circumvent the Tor1 effects on *BRG1* expression. Unexpectedly, we found that Brg1 could bind to several promoters of hypha-specific genes at 37°C independent of rapamycin (Figures 4A and S7). Interestingly, Brg1 could not bind to its own promoter without rapamycin at 37°C (Figure 4B). Nrg1 was not responsible for the Tor1-regulated Brg1 binding, as the association of Brg1 to its own promoter was still rapamycin dependent in the *nrg1* mutant (Figure 4B). Our data show that reduced Tor1 signaling is required for the positive feedback activation by Brg1 on its own promoter. Tor1 signaling does not regulate Brg1 nuclear localization, its interaction with Hda1, or its binding to the UAS regions of hypha-specific genes.

### Ectopically expressed Brg1 does not bind to the promoters of hypha-specific genes in yeast cells

Nrg1 can bind to promoters of hypha-specific genes in yeast cells but not in hyphal cells [18]. Brg1 binds to the promoters in hyphal cells to sustain hyphal development through Hda1 recruitment (Figure 2B). However, the lack of promoter-bound Brg1 in yeast cells could be due to low levels of Brg1 protein, as *BRG1* expression is activated during hyphal elongation (Figure 3). To examine whether Brg1 can bind the promoters of hypha-specific genes in yeast cells, ChIP was performed in yeast cells carrying ectopically expressed Brg1. As shown in Figure 4A, Brg1 could not bind to the UAS region at the *HWP1* promoter in yeast cells at 25°C (Figure 4A). In contrast, Brg1 could bind to the *HWP1* promoter at 37°C (Figure 4A), a condition where Nrg1 was temporarily removed [18]. Similarly, Brg1 could also bind to the UAS regions of *ECE1* and *ALS3* promoters at 37°C, but not at 25°C (Figure S7). Growth at 37°C was required to clear Nrg1 because Brg1 could bind to the promoters in the *nrg1* mutant regardless of temperature (Figure 4A). Therefore, promoters of hypha-specific genes are not accessible to Brg1 in yeast cells.

### Ectopically expressed Brg1 can sustain hyphal growth in the absence of rapamycin

Since reduced TOR signaling sustains hyphal growth through the activation of *BRG1* expression, we examined whether overexpression of *BRG1* could bypass the requirement of rapamycin in sustained hyphal development. Ectopic expression of *BRG1* in a wild-type strain could sustain hyphal growth at 37°C independent of rapamycin (Figure 4C). This functional assay further supports our conclusion that the Tor1 pathway does not directly regulate hypha-specific transcription.

We also observed that overexpressing Brg1 at 25°C could not induce hyphal development (Figure 4C), consistent with the result that Brg1 could not bind to promoters of hyphal genes and *BRG1* in yeast phase (Figure 4AB). Although ectopic expression of Brg1 did not induce robust hyphal development after 6 hours, a few cells became elongated. The low percentage of elongated cells from ectopic Brg1 expression in yeast condition was in contrast to its ability to sustain hyphal development in the absence of rapamycin. Therefore, Brg1's function is to sustain hyphal development and Tor1 signaling controls hyphal elongation through the regulation of *BRG1* expression.

### Nucleosome positions determine promoter accessibility to Nrg1 and Brg1 in yeast and hyphal states

**Figure 5:**
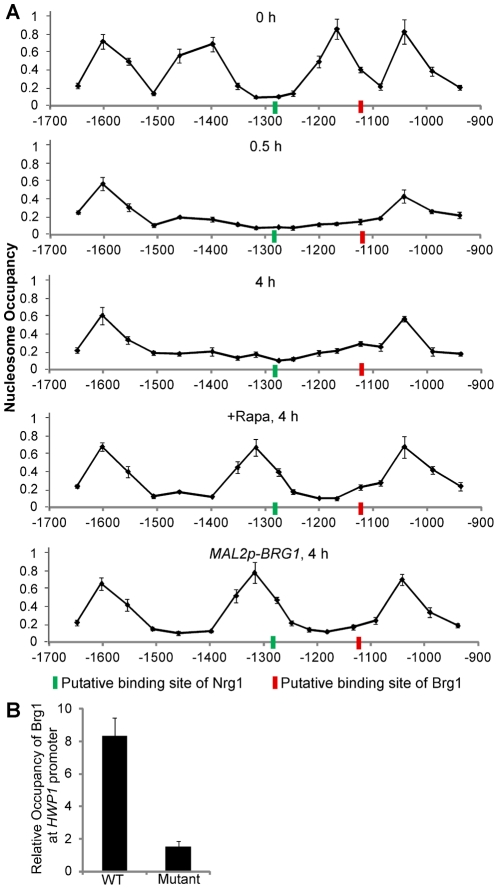
Nucleosome mapping analysis at the UAS region of the *HWP1* promoter indicates different promoter accessibility to Nrg1 and Brg1 in yeast and hyphal cells. Overnight cultures of wild type (SN250) cells were diluted 1∶100 fold into YPD medium in the presence or absence of 10 nM rapamycin at 37°C. Cells of wild type carrying *MAL2p-BRG1* (HLY3636) were diluted 1∶100 fold into YPMaltose medium at 37°C. Cells were collected at indicated times and conditions for nucleosome mapping analysis. PCR primer pairs were designed to generate ∼100 bp fragments, with ∼60 bp overlapping and a 40 bp gap between neighboring PCR fragments. The x-axis represents the midpoints of qPCR reactions. The signals from genomic DNA from each sample for each primer pair were used for normalization. Data show the average of three independent qPCR experiments with error bars representing the SEM. Putative binding sites of Nrg1 (−1284∼−1279) and Brg1 (−1130∼−1125) in the UAS region of the *HWP1* promoter are marked. (B). The predicted Brg1 binding motif in the UAS region of the *HWP1* promoter is required for Brg1 binding. An overnight culture of HLY4079 (carrying both the WT *HWP1* promoter and a copy of the *HWP1* promoter mutated at the Brg1 binding site) was inoculated at a 1∶100 dilution into YPD medium in the presence of 10 nM rapamycin at 37°C for 4 h. Primers 21 and 22 were used to quantitate the binding of Brg1 to the WT *HWP1* promoter over no tag control, and primers 21 and 23 were used to quantitate the binding of Brg1 to the mutated *HWP1* promoter over no tag control.

Nucleosomes are generally inhibitory to transcription factor binding, and changes in nucleosome position are associated with changes in gene expression by affecting the accessibility of transcription factors to promoters [31], [32]. We suspected that changes in nucleosome positioning during yeast-to-hypha development determined the differential promoter accessibility to Nrg1 and Brg1 in yeast and hyphal cells. To test this hypothesis, we mapped nucleosome positions around the UAS region of the *HWP1* promoter with cells in yeast phase, during hyphal initiation, during hyphal elongation with and without rapamycin, or with ectopic expression of Brg1 (Figure 5A). We observed a nucleosome free region (NFR) in the middle of the UAS region on the *HWP1* promoter in yeast phase cells from the starting culture at 0 hours. After 0.5 hours of hyphal induction in YPD at 37°C, the two nucleosomes surrounding the NFR disappeared, leaving a large region without nucleosomes. Interestingly, the two nucleosomes stayed off in the hyphal cells after 4 hours of growth in YPD at 37°C, a condition in which *BRG1* expression is low. During sustained hyphal growth in the presence of rapamycin for *BRG1* expression, or when Brg1 was ectopically expressed, a new nucleosome appeared in the middle of the UAS region, leaving two NFRs around the nucleosome. Our nucleosome mapping experiments demonstrated changes in nucleosome positioning at the UAS region of the *HWP1* promoter during yeast-to-hypha development: from two nucleosomes in yeast phase, to no nucleosomes in the initiation phase, to one nucleosome in the hyphal phase. The observed changes in nucleosome occupation at the UAS region are consistent with our previous findings. Removal of Nrg1 during hyphal induction leads to rapid histone disassembly on UAS regions of hyphal genes [18], which correlates to the dissociation of the two nucleosomes around the NFR in the UAS of the *HWP1* promoter. The formation of a new nucleosome during hyphal elongation requires Brg1 binding and the recruitment of Hda1, consistent with the finding that Hda1 at the promoters leads to eviction of the NuA4 acetyltransferase module and chromatin remodeling [18].

DNA sequence analysis for potential Nrg1 and Brg1 binding motifs in the UAS region of the *HWP1* promoter identified one Nrg1 binding site and one Brg1 binding site. Interestingly, the Nrg1 binding site is located in the NFR in yeast cells; therefore it should be accessible to Nrg1 binding. However, the Brg1 binding site is occupied by a nucleosome, making it not accessible to Brg1 (Figure 5A, 0 hour). During sustained hyphal development, the Nrg1 binding site is occupied by a nucleosome and becomes inaccessible whereas the Brg1 binding site is exposed in NFRs (Figure 5A, +Rapa or *MAL2p-BRG1*). Both binding sites are accessible during the hyphal initiation phase (Figure 5A, 0.5 hour). Our data suggest that changes in nucleosome positioning during the yeast-to-hypha development underlie the differences in promoter accessibility to Nrg1 and Brg1. Consistent with the concept that nucleosomes are inhibitory to transcription factor binding, Nrg1 binding motifs are found in nucleosome free regions upstream of repressed genes by genome-wide nucleosome mapping of the yeast form of *C. albicans* [32]. Furthermore, DNA sequence analysis of the intergenic regions upstream of *BRG1*, *HWP1*, *ALS3* and *ECE1* identified many Brg1 and Nrg1 binding motifs, but only the UAS regions are bound by Nrg1 in yeast cells and by Brg1 in hyphal cells (Figures S6 and S8ABC). Interestingly, all UAS regions of these promoters contain both Nrg1 and Brg1 binding motifs. In addition, Brg1 also binds to the promoters of two hypha-specific regulatory genes, *UME6* and *HGC1*, during hyphal elongation (Figure S9AB). Consistent with other hyphal regulated genes, Brg1-bound regions on the two promoters contain both Nrg1 and Brg1 binding sites within the distance of a nucleosome, and regions with only Brg1 binding sequence are not bound by Brg1 (Figure S9AB). All these support the model that dynamic changes in nucleosome positioning on promoters of hypha-specific genes during the yeast-to-hypha transition lead to different accessibilities of these promoters to Nrg1 and Brg1.

Our results infer that Brg1 mediates its effects by binding directly to its binding sites at nucleosome free regions on the promoters of many hypha-specific genes. To examine the functionality of the putative Brg1 binding site in the UAS region for *HWP1*, we replaced the Brg1 binding sequence AGGTAA at −1130 to −1125 on the *HWP1* promoter with CTAGCC. The mutated *HWP1* promoter was introduced to a wild-type *C. albicans* strain, generating a strain that carries both the wild-type *HWP1* promoter and a copy of the *HWP1* promoter mutated at the putative Brg1 binding site. ChIP of Brg1 under a condition of hyphal elongation showed the mutated *HWP1* UAS region was not associated with Brg1 in comparison to the wild type copy of the *HWP1* promoter in the same cells (Figure 5B). Therefore, the predicted Brg1 binding site in the UAS region (−1130 to −1125) is directly responsible for Brg1 recruitment.

### Ume6 functions after Brg1 in a positive feedback regulation of hypha-specific genes to sustain hyphal development

**Figure 6:**
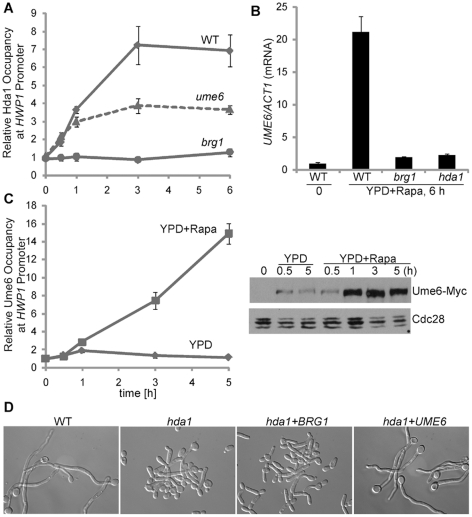
Ume6 functions downstream of Brg1 and Hda1 in hyphal maintenance. (A). Ume6 is not essential for promoter recruitment of Hda1 during hyphal elongation. ChIP of Hda1-Myc in wild type, *brg1*, and *ume6* mutants in YPD with 10 nM rapamycin at 37°C. (B). *UME6* expression requires Brg1 and Hda1. Expression levels were quantified by qRT-PCR and normalized with *ACT1*. All data show the average of three independent qRT-PCR or qPCR experiments with error bars representing the SEM. (C). Ume6 binds to hyphal promoters during hyphal elongation. ChIP and Western analysis of Ume6 in wild type cells (HLY4078) in indicated media. (D). Ectopic expression of *UME6*, but not *BRG1*, restores filamentous growth in the *hda1* mutant. *hda1* cells transformed with pBES116 (V), MAL2p-MYC-BRG1 (HLY4077) or MAL2p-UME6-MYC (HLY4076) were grown in YEPMaltose medium with 5 nM rapamycin for cell morphology.

Ume6 has been shown to control the level and duration of hypha-specific genes and is important for hyphal elongation [9]–[11]. To address the functional relationship between Ume6 and Hda1 recruitment by Brg1 in hyphal maintenance, we performed a time course ChIP experiment of Hda1-Myc in *brg1* and *ume6* mutants. Unlike Brg1, Ume6 was not essential for promoter recruitment of Hda1, although levels of promoter-associated Hda1 decreased in the *ume6* mutant compared to that in the wild-type strain (Figure 6A). Similar to other hypha-specific genes, *UME6* expression is dependent on Brg1 and Hda1 (Figure 6B). However, protein levels of Ume6 increased much slower and reached a much lower level during hyphal induction in the absence of rapamycin than expected (Figure 6C). Unstable *UME6* transcript or protein likely made Ume6 level sensitive to the level of *UME6* expression and rendered Ume6 protein level more dependent on Brg1 function than other proteins specifically expressed in hyphal cells. We then examined whether overexpression of *UME6* could bypass the requirement of Hda1 in hyphal elongation. As shown in Figure 6D, constitutively expressed Ume6, but not Brg1, restored the hyphal growth defect in *hda1* mutant cells. This result strongly suggests that expressing *UME6* is a major function of Brg1-mediated chromatin remodeling by Hda1 during hyphal elongation. We further showed that Ume6 binds directly to the UAS region of the *HWP1* promoter (Figure 6C). Interestingly, levels of promoter-bound Ume6 peaked later than Brg1 during hyphal induction (Figure 6C vs. 2B). Based on our data, we suggest that Ume6 functions directly on the promoters of hypha-specific genes after Brg1 as a built-in positive feedback for sustained hyphal development.

## Discussion

Here we report the identification of Brg1, by a forward genetic screen, as the transcription factor that recruits Hda1 to promoters of hypha-specific genes under reduced Tor1 signaling for sustained hyphal development. Many signaling pathways and transcription factors play a role in hyphal morphogenesis in *C. albicans*. This is evident from the vast literature on this topic and number of genes implicated in hyphal morphogenesis [33]. In addition, large-scale mutant screens have also identified many regulators of hyphal morphogenesis including Brg1 [27], [28]. A challenge is how to dissect the complicated regulatory network and understand the underlying molecular mechanisms for each regulator or pathway in hyphal development. Our recent study suggests that hyphal development undergoes two phases of regulation in promoter chromatin: initiation and elongation [18]. Nrg1 is temporally removed upon activation of the cAMP pathway during initiation; Hda1 is recruited to the promoters of hypha-specific genes under reduced Tor1 signaling during elongation. With this as our framework, we find that, among 165 putative transcription factor mutants, 7 mutants are defective in germ tube formation, and 47 mutants in hyphal elongation to different extents. Among the 47 mutants, 8 are still defective in the presence of rapamycin. Some of the 8 regulators may act downstream of Tor1 signaling. Out of the 8 mutants, we further narrowed it down to *brg1* as the only mutant defective in the promoter recruitment of Hda1. Consistent with the mutant phenotype, we find that Brg1 interacts with Hda1. Furthermore, it binds to the promoters of hypha-specific genes during hyphal elongation in a rapamycin-dependent manner. Therefore, by separating defects in hyphal initiation vs. elongation, and by examining whether rapamycin could bypass the defects, as well as by using Nrg1 protein level and Hda1 promoter recruitment as readouts, we are able to identify one, from many mutants defective in hyphal development, that is specifically involved in Hda1 promoter recruitment. We also find that *ahr1* and *rob1* mutants are partially defective in hyphal development due to impaired down-regulation of Nrg1. Ahr1 has been previously shown to function in hyphal development [27], [34]. It is a zinc-finger transcription factor that recruits Mcm1 to promoters of genes involved in biofilm formation, filamentous growth, etc. including *EFG1* and *NRG1* [34]. Based on our data and Ahr1 target genes [34], Ahr1 likely affects hyphal development through the regulation of *EFG1* and/or *NRG1* expression. Our forward genetic screening demonstrates that this scheme of mutant analyses can provide a framework for further characterization of other mutants defective in hyphal development.

*BRG1* expression is a sensitive output of Tor1 signaling in *C. albicans*. Sustained hyphal development requires only a sub-growth inhibitory level of 5–10 nM rapamycin [18]. This is 10-fold lower than the concentration required for nuclear localization of the GATA transcription factors Gln3 and Gat1 in *S. cerevisiae* [23]. Similarly, we have observed nuclear localization of Gln3 in *C. albicans* only in the presence of 100 nM rapamycin, but not at 10 nM (our unpublished data). In contrast to Gln3, Brg1 nuclear localization is not regulated by rapamycin. Instead, *BRG1* expression requires the presence of 5–10 nM rapamycin, making it a sensitive readout of Tor1 signaling. In addition to reduced Tor1 signaling, activation of *BRG1* expression also requires the removal of Nrg1 inhibition. This is different from hypha-specific genes where lack of Nrg1 is sufficient for their expression. Therefore, *BRG1* expression uses an AND gate logic gating for signal integration. This type of logic gating is also seen in other GATA factor regulated gene expression. For example, the expression of nitrate and nitrite reductase in *Aspergillus nidulans* requires both the presence of nitrate and the absence of ammonium and glutamine [35]. Nrg1 and Brg1 act directly on the *BRG1* promoter. Similar to hyphal specific promoters, Nrg1 dissociates from the *BRG1* promoter during hyphal initiation and subsequently, Brg1 binds to the promoters during hyphal elongation in a rapamycin-dependent manner. Brg1 protein is not a direct target of the Tor1 pathway because Brg1 association with hypha-specific promoters is independent of rapamycin and ectopic expression of Brg1 can sustain hyphal elongation in the absence of rapamycin. Therefore, Tor1 function in hyphal development is through the transcriptional regulation of *BRG1*. Experiments are underway to identify the additional regulators of *BRG1* expression.

**Figure 7:**
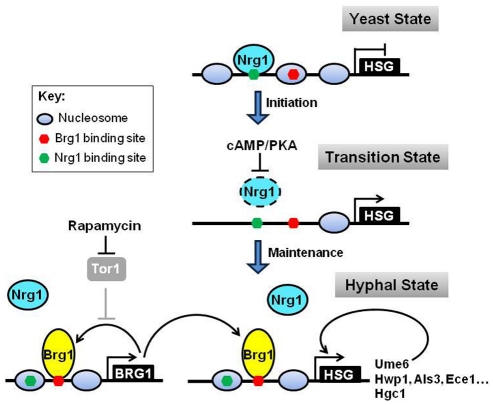
A schematic diagram depicting temporal dynamic regulation of promoter chromatin during the yeast-to-hypha transition. Hyphal development undergoes sequential changes in nucleosome positioning from the yeast state, to a transition state, and then to the hyphal state by changing promoter accessibility to Nrg1 and Brg1. Black lines represent active regulatory relationships; gray lines represent relationships that are inactive; dashed lines represent relationships that are not yet determined. Dashed circles represent degraded proteins. HSG, hypha-specific genes.

The position of nucleosomes in a gene promoter impacts the accessibility of transcription factors to their DNA-binding sites. Ectopically expressed Brg1 binds promoters of hypha-specific genes only in hyphal cells, but not in yeast cells. Conversely, Nrg1 binds to the promoters in yeast cells, but not in hyphal cells. By mapping nucleosome positions during yeast-to-hypha development at the UAS region of the *HWP1* promoter, we find dynamic changes in nucleosome positioning at the UAS region. The observed change in nucleosome positioning provides a mechanism for differential accessibility to Nrg1 and Brg1 in yeast and hyphal cells. Other hypha-specific promoters, including *UME6* and *HGC1* as well as *BRG1*, likely have different nucleosome positioning in yeast and hyphae similar to *HWP1* as differential Brg1 and Nrg1 binding is observed for all hypha-specific promoters examined. Furthermore, Brg1 bound regions on the examined promoters all have both Brg1 and Nrg1 binding sites within the distance of one nucleosome. We propose that hyphal development undergoes sequential changes in nucleosome positioning from the yeast state, to a transition state, and then to the hyphal state (Figure 7). In the yeast state Nrg1 binding sites are in a nucleosome free region and accessible, whereas Brg1 bindings sites are occupied by nucleosomes and therefore not accessible. Upon activation of the cAMP/PKA pathway when cells are inoculated into fresh media at 37°C, Nrg1 is temporarily removed, leading to rapid nucleosome disassembly at the UAS regions [18]. During the transition state, binding sites for both Nrg1 and Brg1 are accessible, providing a time window for Brg1 accumulation and binding to promoters before Nrg1 returns. In the hyphal state, nucleosomes reposition through Brg1-mediated Hda1 recruitment and chromatin remodeling, thus the Nrg1 binding sites are not accessible. The precise positions of Brg1 binding sites relative to nucleosomes in the hyphal state may vary among the promoters as they differ in levels or extent of expression during hyphal development. This effect of nucleosome positions on transcription factor binding and transcription induction is convincingly demonstrated by the Pho5 system in *S. cerevisiae* [36]. On the other hand, nucleosome occupancy is dynamic and is determined by a balance of assembly and disassembly activities. When highly expressed, Brg1 can compete with nucleosomes and convert some cells from the yeast to hyphal state. This is a slow and occasional event, consistent with the existence of a few elongated cells in a culture of mostly yeast cells when Brg1 is expressed from the *MAL2* promoter under yeast growth conditions. A change in nucleosome positioning during yeast-to-hypha development provides a molecular mechanism for temporally linked regulation of promoter chromatin. It also provides a mechanism for plasticity in hyphal development and explains cell-to-cell variation among a cell population, which is important for adaptation and survival of unicellular microorganisms in stress conditions.

The function of Brg1 in hyphal development is to recruit Hda1 to the UAS regions of hypha-specific promoters. Hda1 in turn remodels promoter chromatin by deacetylating a subunit of the NuA4 histone acetyltransferase module, leading to the occlusion of Nrg1 binding sites [18]. We have also shown that hyphal induction either in the presence of rapamycin, a condition for *BRG1* expression or with the ectopic expression of Brg1, leads to nucleosome repositioning. This function of Brg1 in chromatin remodeling in likely common as other GATA factors have also been shown to play a role in nucleosome rearrangements [35], [37]–[39]. The ultimate function of Brg1 in chromatin rearrangement during hyphal development is to remove Nrg1 inhibition. This provides another layer of Nrg1 regulation, in addition to regulating its expression and stability. The additional layer of regulation provides *C. albicans* with a flexible response to nutrition and various growth environments.

One key downstream target of the Brg1-mediated chromatin regulation is *UME6*. Brg1 binds upstream of *UME6*, and *UME6* expression depends on Brg1 and Hda1. Furthermore, ectopic expression of Ume6 completely bypassed the requirement of Brg1 or Hda1. In this sense, hypha-specific expression of some genes could be regulated indirectly by Brg1 through the control of the level of Ume6. Our results demonstrate that Ume6 functions after Brg1 and Hda1 as a built-in positive feedback regulation of the hyphal transcriptional program to sustain hyphal development, consistent with its reported functions [9]–[11]. We suggest that the yeast-to-hypha transition is controlled by a feed-forward loop of regulations by Nrg1, Brg1, and Ume6. This temporal regulatory model is in agreement with delayed hyphal development by ectopic expression of Ume6 in yeast growth conditions [10]. Brg1 plays a critical function as a mid-regulator in this feed-forward loop. It provides *C. albicans* with a means to sense and integrate multiple nutritional and environmental signals for control of its morphological program that is essential for its pathogenesis. Considering that misregulation of either Nrg1 or Ume6 level causes altered virulence [9], [40], our identification of Brg1 as an output of the Tor1 signaling that regulates Nrg1 binding and Ume6 expression should provide a better understanding of the molecular mechanisms that control the yeast-hyphal development in its hosts, and how *C. albicans* senses and integrates multiple nutritional and environmental signals in the host for its adaption to exist as both a commensal and pathogen.

## Materials and Methods

### Screening of transcription factor mutants defective in hyphal elongation

165 putative transcription factor mutants [27] and the wild-type reference strain SN250 were grown overnight in liquid YPD at 30°C. 15 mutants grew as elongated pseudohyphae and they were excluded from further analysis. The remaining 150 mutants and wild type cells were diluted at 1∶100 to YPD+10% Serum at 37°C for 1 h for a germ tube formation screen. 14 mutants were defective in germ tube formation. Among them, 7 completely blocked germ tube formation and were excluded. For mutants defective hyphal elongation, the 143 remaining mutants were grown in Lee's medium for 6 h at 37°C, and 47 mutants were defective in hyphal elongation. Among these 47 mutants, 39 mutants could be restored in hyphal elongation by rapamycin in YPD at 37°C. Only 8 transcription factor mutants were defective in response to rapamycin induced hyphal maintenance.

### Plasmid and strain construction

The *C. albicans* strains used in this study are listed in Table S1. Primer sequences are listed in Table S2. The 8 transcription mutants defective in rapamycin induced hyphal elongation and UZ43 [11] were streaked on 5-fluoro-orotic acid-containing medium to generate Ura^–^ strains for these mutants. pPR673-HDA1 was digested with *BamH*I to target the integration of the plasmid into the *HDA1* locus to express Hda1-13Myc in these mutants. A 1.2-kb PCR product (primers 1 and 2) containing the C-terminal *BRG1* coding region was inserted into the *BamH*I-*Mlu*I sites of pPR671 and pPR673 [30]. The resulting plasmids were digested with *Spe*I for target integration into the *BRG1* loci to express Brg1-13Myc. The pMAL2-MYC-BRG1 plasmid was constructed by amplifying *BRG1* (primers 3 and 4) to replace *HGC1* from the pMAL2-MYC-HGC1 plasmid [41]. The resulting plasmid was digested with *Asc*I to target integration into the *ADE2* locus to express Brg1-13Myc. A 1.3-kb PCR product (primers 5 and 6) containing the C-terminal *HDA1* coding region was digested with *Bgl*II and *Mlu*I to replace *CPH1* from the pACT1-CPH1-TAP plasmid (our unpublished data). The resulting plasmid was digested with *BamH*I to target the integration of the plasmid into the *HDA1* locus in HLY3636 to express Hda1-TAP. The pMAL2-UME6-13MYC plasmid was constructed by amplifying *UME6* (primers 7 and 8) to replace *NRG1* from the pMAL2-NRG1-MYC plasmid. The resulting plasmids were digested with *Asc*I for target integration into the *ADE2* locus in HLY4032 to express Ume6-13Myc. To construct a mutant HWP1 promoter with a site-specific mutation at the predicted Brg1 binding site in the UAS region, the AGGTAA sequence at −1130 to −1125 upstream of *HWP1* was replaced by CTAGCC. Two pairs of primers (primers 17 and 18, 19 and 20) were used to PCR amplify overlapping *HWP1* promoter fragments with the mutation in the overlapping region. The resulting PCR products were purified and mixed as templates for another round of PCR amplification using the primers 17 and 20, which produced the full-length mutated *HWP1* promoter sequence. The resulting PCR product was cloned into the KpnI-ClaI site of the plasmid pHL471 [42] to express GFP, and was confirmed by DNA sequencing. The resulting plasmid was digested with *Bgl*II within the *HWP1* promoter region (from −1465 to −1460) for integration into the endogenous *HWP1* promoter locus. Primers 21 and 22 were used to quantitate the binding of Brg1 to the wild type *HWP1* promoter, and primers 21 and 23 were used to quantitate the binding of Brg1 to the mutated *HWP1* promoter.

### Chromatin immunoprecipitation

Chromatin immunoprecipitation was performed as described with modifications [30]. DNA was sheared by sonication six times for 20 seconds at high power on a Bioruptor (diagenode) with 40 second intervals on ice. 10 µl of anti-Myc (SC-789, Santa Cruz) antibodies were used for ∼4 mg of chromatin proteins in an immunoprecipitation volume of 200 µl.

### Immunoprecipitation

Protein extraction and immunoprecipitation were performed as described previously (Cao *et al.*, 2006) with modifications. Protein extract containing 10 mg protein were subjected to immunoprecipitation using 60 µl of immunoglobulin G (IgG) agarose bead slurry (Sigma), which was preincubated once with 0.2 mg/ml sheared salmon sperm DNA, 0.5 mg/ml bovine serum albumin in phosphate-buffered saline. Proteins were separated by 8% SDS-polyacrylamide gel electrophoresis and transferred to a polyvinylidene difluoride membrane (Hybond; GE Healthcare). After blocking in 3% skim-milk powder in Tris-buffered saline/0.05% Tween 20, a peroxidase-conjugated anti-c-Myc antibody (Roche) was used to probe for Myc-tagged proteins, which were then detected using the enhanced chemiluminescence system (Pierce Chemical. Rockford, IL).

### Quantitative PCR expression analysis

Methods for RNA isolation were carried out as previously described [43]. 10 µg of total RNA was DNase-treated at 37°C for 1 h using the DNase-free kit (Qiagen), cDNA was synthesized using the SuperScript II Reverse Transcriptase kit (Invitrogen), and qPCR was done using the iQ SYBR Green Supermix (Bio-Rad) using the primers 9 and 10 for *BRG1*, primers 11 and 12 for *ACT1* and primers 13 and 14 for *UME6*.

### Nucleosome mapping

For the MNase assay, we used the protocol described as previously described [44], [45]. In brief, we harvested 5×10^7^ cells of each sample, and washed in 1 ml water. Then we resuspended the cells in 1 ml of sphaeroplasting solution (1 M sorbitol, 0.5 mM 2-mercaptoethanol, 0.18 mg/ml zymolyase), and incubated at room temperature for 5 min with gentle stirring. We harvested the cells, washed it in 1 ml of 1 M sorbitol, then resuspended the pellet in 500 µl of digestion buffer (1 M sorbitol, 50 mM NaCl, 100 mM Tris-Cl [pH 7.4], 5 mM MgCl_2_, 1 mM CaCl_2_, 1 mM 2-mercaptoethanol, 0.5 mM spermidine, 0.075% NP-40, micrococcal nuclease with a final concentration 1–10 U/ml) for 8 min at 37°C. After terminating the MNase digestion by adding 50 µl quench buffer (250 mM EDTA, 5% SDS), DNA was extracted with phenol/chloroform, and then we proceeded with the qPCR analysis. The PCR product was designed to be ∼100 bp, and the neighboring PCR primers are ∼40 bp apart. In the nucleosome map, the x-axis represents the midpoint of the PCR product. The signal of each primer pair obtained from genomic DNA from each sample was used for normalization. The values of nucleosome occupancy at −5188 on the *WOR1* promoter, which is not regulated by the yeast-hypha transition, were set to be 1.00.

## Supporting Information

Figure S1 **Brg1 is required for hyphal elongation and promoter recruitment of Hda1 in serum-containing media.** (A). Wild type and *brg1* mutant cells were inoculated into YPD+10% serum medium at 37°C and grown for 4 h. (B). Wild type and *brg1* mutant cells carrying Hda1-Myc were inoculated into YPD+10% serum at 37°C and grown for 3 h. ChIP DNA was quantitated with primers at the UAS region of *HWP1*.
(TIF)

Figure S2 **Recruitment of Hda1 to promoters of** ***ALS3*** **and** ***ECE1*** **in the presence rapamycin is Brg1 dependent.** Wild type and *brg1* mutant cells carrying Hda1-Myc were inoculated into YPD+10 nM rapamycin medium at 37°C and grown for 6 h, as described in Figure 1. ChIP DNA was quantitated with primers at the UAS regions of *ALS3* and *ECE1* using primers described in [30], and the region −672∼−447 of the *ALS3* promoter that contains a GATA factor binding site.
(TIF)

Figure S3 **Brg1 is constitutively localized in the nucleus.** Wild-type strain expressing Myc-Brg1 under the *MAL2* promoter (HLY3636) was grown in YPD medium at 37°C in the presence or absence of 10 nM rapamycin. Cells were fixed at 3 h after inoculation and processed for indirect immunofluorescence with a method as described [41] with 9E10 mouse antibodies and FITC-conjugated secondary antibodies. DNA was stained with DAPI. An untagged control (SC5314) was included.
(TIF)

Figure S4 **Western analysis of Brg1 in YPD medium with or without rapamycin at 25°C or 37°C.** (A) Wild-type cells carrying Brg1-Myc (HLY4059) were diluted into the indicated media and conditions, and cells were collected at 0 min, 1 h, 3 h, and 5 h for Western analysis. (B) Brg1 protein stability is not regulated by rapamycin. Western of wild-type cells carrying Myc-Brg1 under the *MAL2* promoter inoculated from overnight culture into fresh YPD medium at 25°C or 37°C in the presence or absence 10 nM rapamycin.
(TIF)

Figure S5 **Constitutively expressed** ***NRG1*** **blocks** ***BRG1*** **activation.** Cells of wild type or the *nrg1* mutant carrying MAL2p-Nrg1-Myc, from overnight cultures in YEP Maltose at 30°C, were inoculated at 1∶20 dilution into YEP Maltose at 37°C in the presence of 10 nM rapamycin and grown for 1 h. *BRG1* mRNA levels were determined by RT-PCR as Fig. 3A.
(TIF)

Figure S6 **ChIP of Nrg1 and Brg1 at different regions of the** ***BRG1*** **promoter.** Wild-type cells carrying Nrg1-Myc or Brg1-Myc were grown in YPD medium at 25°C or YPD+10 nM rapamycin at 37°C, respectively, for 6 h. SC5314 was used as a no tag control. The enrichment over that of untagged controls is shown. Locations of specific sequence elements are marked (Nrg1 sites [green]: [A/C][A/C/G]CCCT, CCCCT, or CCCTC [20]. Brg1 sites [red]: [A/C]GGTA[C/A] [46]. Positions of primer pairs are indicated in rectangles.
(TIF)

Figure S7 **Ectopically expressed Brg1 could not bind to hyphal promoters in yeast cells.** ChIP of Myc-Brg1 under the *MAL2* promoter in wild-type cells grown in YEPMaltose medium at 25°C or 37°C for 6 h.
(TIF)

Figure S8 **ChIP of Brg1 at different regions of hyphal promoters.** Wild-type cells carrying Brg1-Myc were grown in YPD+10 nM rapamycin at 37°C for 6 h. SC5314 was used as a no tag control. The enrichment over that of untagged controls is shown. Locations of specific sequence elements are marked (Nrg1 sites [green]: [A/C][A/C/G]CCCT, CCCCT, or CCCTC [20]. Brg1 sites [red]: [A/C]GGTA[C/A]) [46]. Positions of primer pairs used for qPCR are indicated and numbered. The UAS region of each promoter is located in the dashed rectangle.
(TIF)

Figure S9 **ChIP of Brg1 at different regions of** ***UME6*** **and** ***HGC1*** **promoters.** Wild-type cells carrying Brg1-Myc were grown in YPD+10 nM rapamycin at 37°C for 6 h. SC5314 was used as a no tag control. Locations of specific sequence elements are marked (Nrg1 sites [green]: [A/C][A/C/G]CCCT, CCCCT, or CCCTC [20]. Gat2 sites [red]: [A/C]GGTA[C/A]) [46]. Positions of primer pairs used for qPCR are indicated and numbered. The enrichment of Brg1 at the indicated regions upstream of *UME6* (A) and *HGC1* (B) over that of untagged control is shown.
(TIF)

Table S1 ***C. albicans*** **strains used in this study.**
(DOC)

Table S2 **Primers used in this study.**
(DOC)
