# Supplementary figures and images for: A GATA Transcription Factor Recruits Hda1 in Response to Reduced Tor1 Signaling to Establish a Hyphal Chromatin State in Candida albicans
Source: PLoS Pathog. 2012 Apr 19;8(4):e1002663. doi: 10.1371/journal.ppat.1002663 (PMC3334898; doi:10.1371/journal.ppat.1002663)

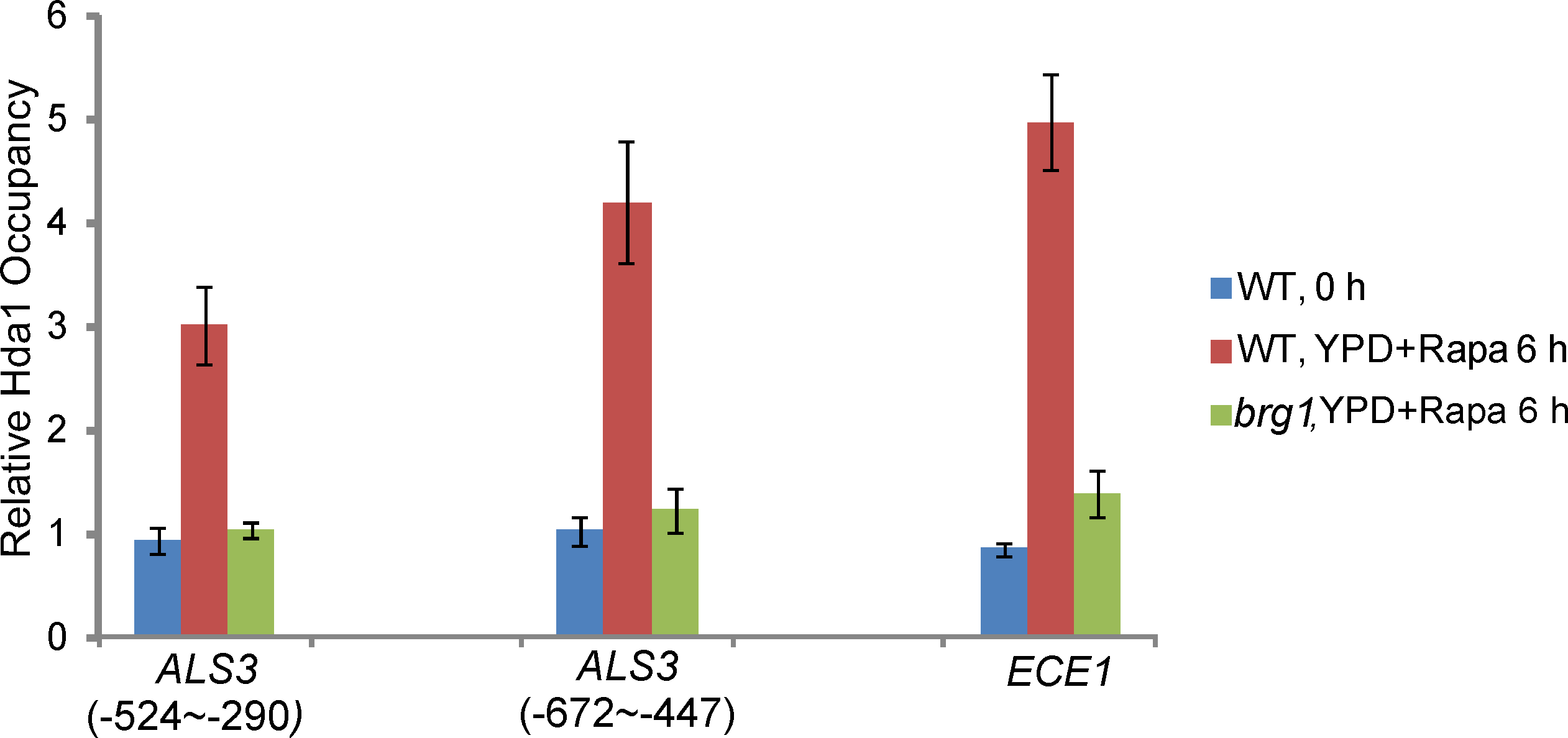

Supplement: Figure S1 — Brg1 is required for hyphal elongation and promoter recruitment of Hda1 in serum-containing media. (A). Wild type and brg1 mutant cells were inoculated into YPD+10% serum medium at 37°C and grown for 4 h. (B). Wild type and brg1 mutant cells carrying Hda1-Myc were inoculated into YPD+10% serum at 37°C and grown for 3 h. ChIP DNA was quantitated with primers at the UAS region of HWP1. (TIF) [file ppat.1002663.s001.tif]

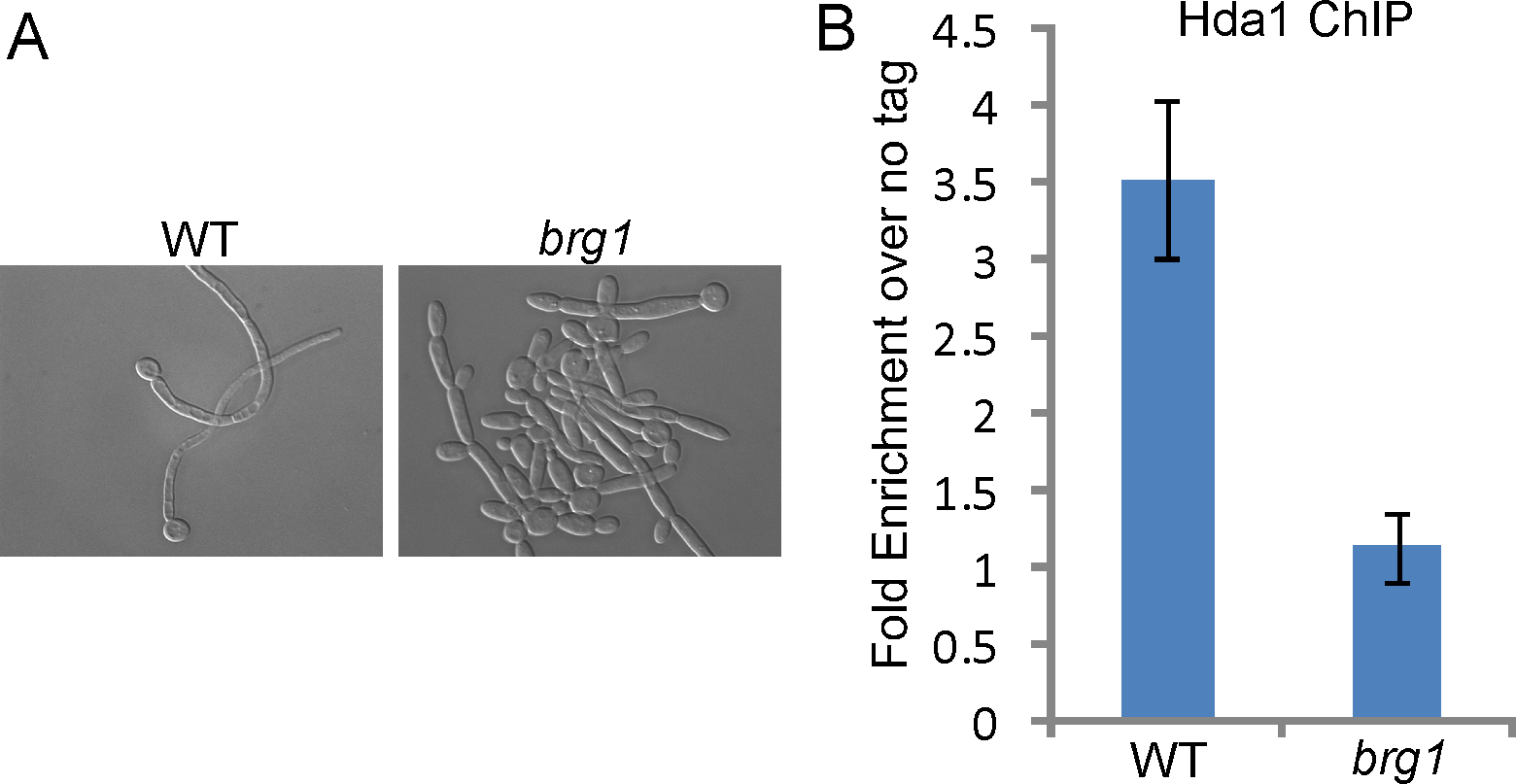

Supplement: Figure S2 — Recruitment of Hda1 to promoters of ALS3 and ECE1 in the presence rapamycin is Brg1 dependent. Wild type and brg1 mutant cells carrying Hda1-Myc were inoculated into YPD+10 nM rapamycin medium at 37°C and grown for 6 h, as described in Figure 1. ChIP DNA was quantitated with primers at the UAS regions of ALS3 and ECE1 using primers described in [30], and the region −672∼−447 of the ALS3 promoter that contains a GATA factor binding site. (TIF) [file ppat.1002663.s002.tif]

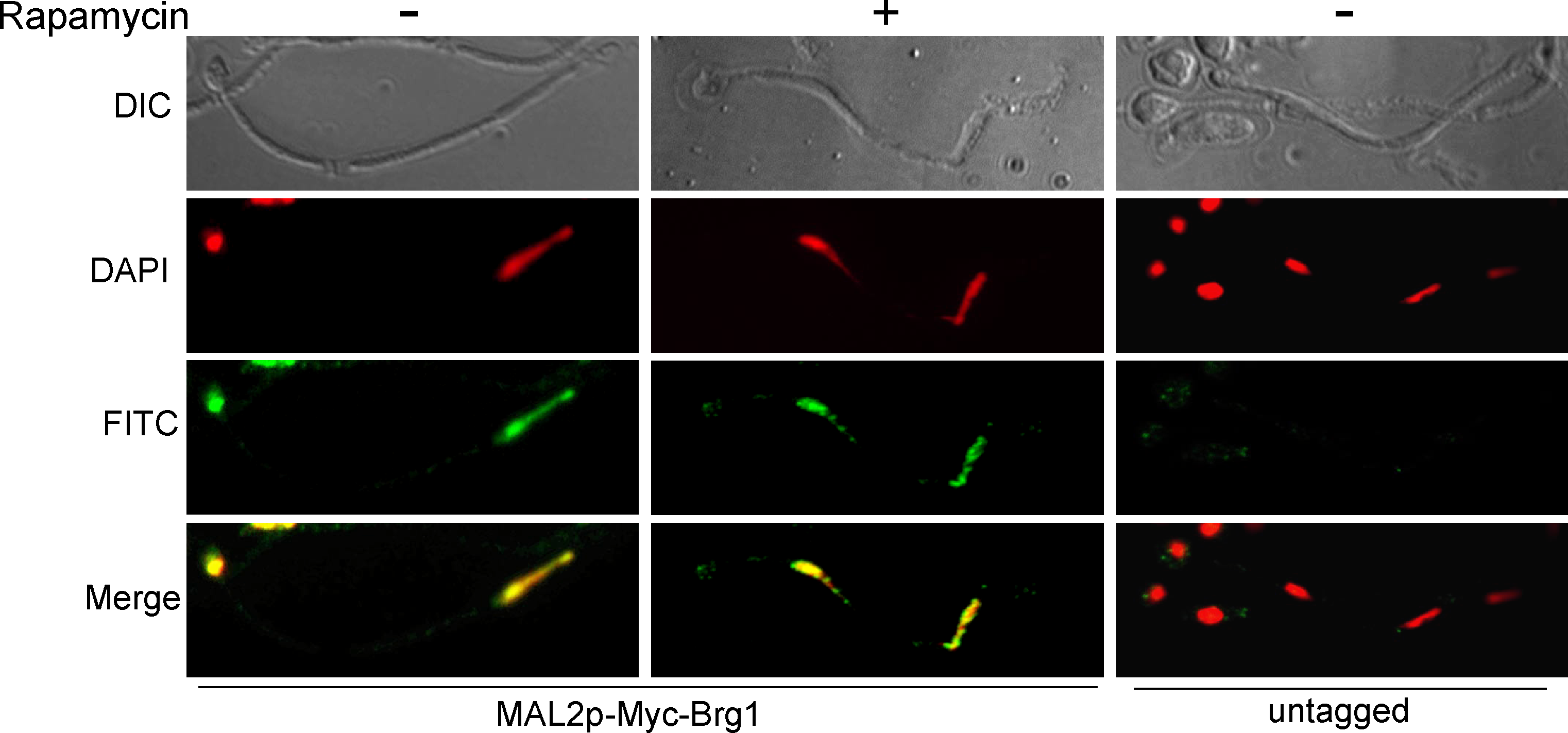

Supplement: Figure S3 — Brg1 is constitutively localized in the nucleus. Wild-type strain expressing Myc-Brg1 under the MAL2 promoter (HLY3636) was grown in YPD medium at 37°C in the presence or absence of 10 nM rapamycin. Cells were fixed at 3 h after inoculation and processed for indirect immunofluorescence with a method as described [41] with 9E10 mouse antibodies and FITC-conjugated secondary antibodies. DNA was stained with DAPI. An untagged control (SC5314) was included. (TIF) [file ppat.1002663.s003.tif]

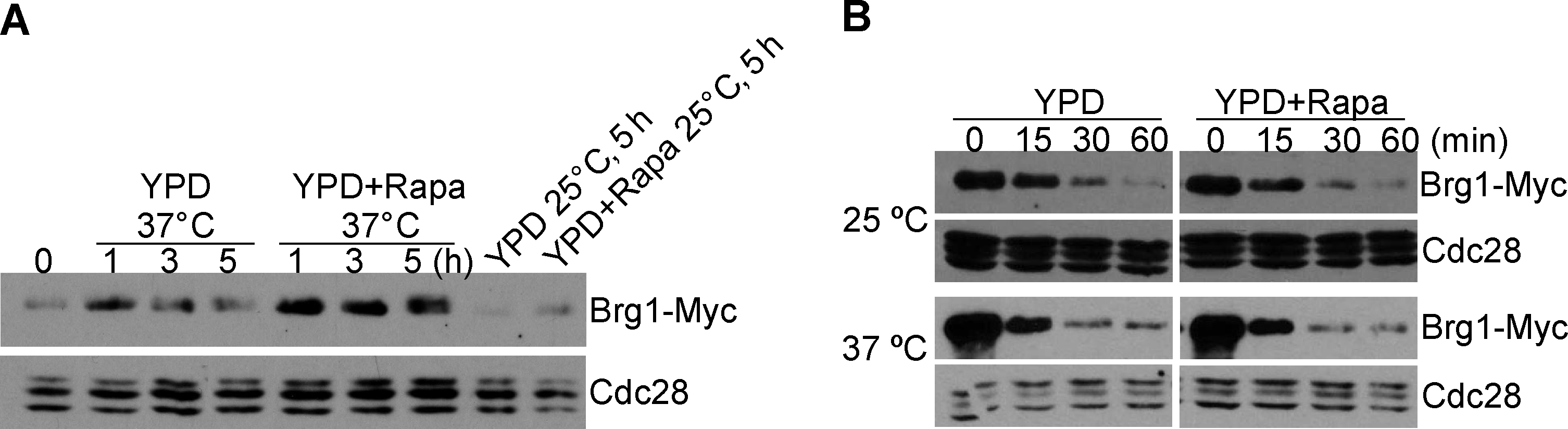

Supplement: Figure S4 — Western analysis of Brg1 in YPD medium with or without rapamycin at 25°C or 37°C. (A) Wild-type cells carrying Brg1-Myc (HLY4059) were diluted into the indicated media and conditions, and cells were collected at 0 min, 1 h, 3 h, and 5 h for Western analysis. (B) Brg1 protein stability is not regulated by rapamycin. Western of wild-type cells carrying Myc-Brg1 under the MAL2 promoter inoculated from overnight culture into fresh YPD medium at 25°C or 37°C in the presence or absence 10 nM rapamycin. (TIF) [file ppat.1002663.s004.tif]

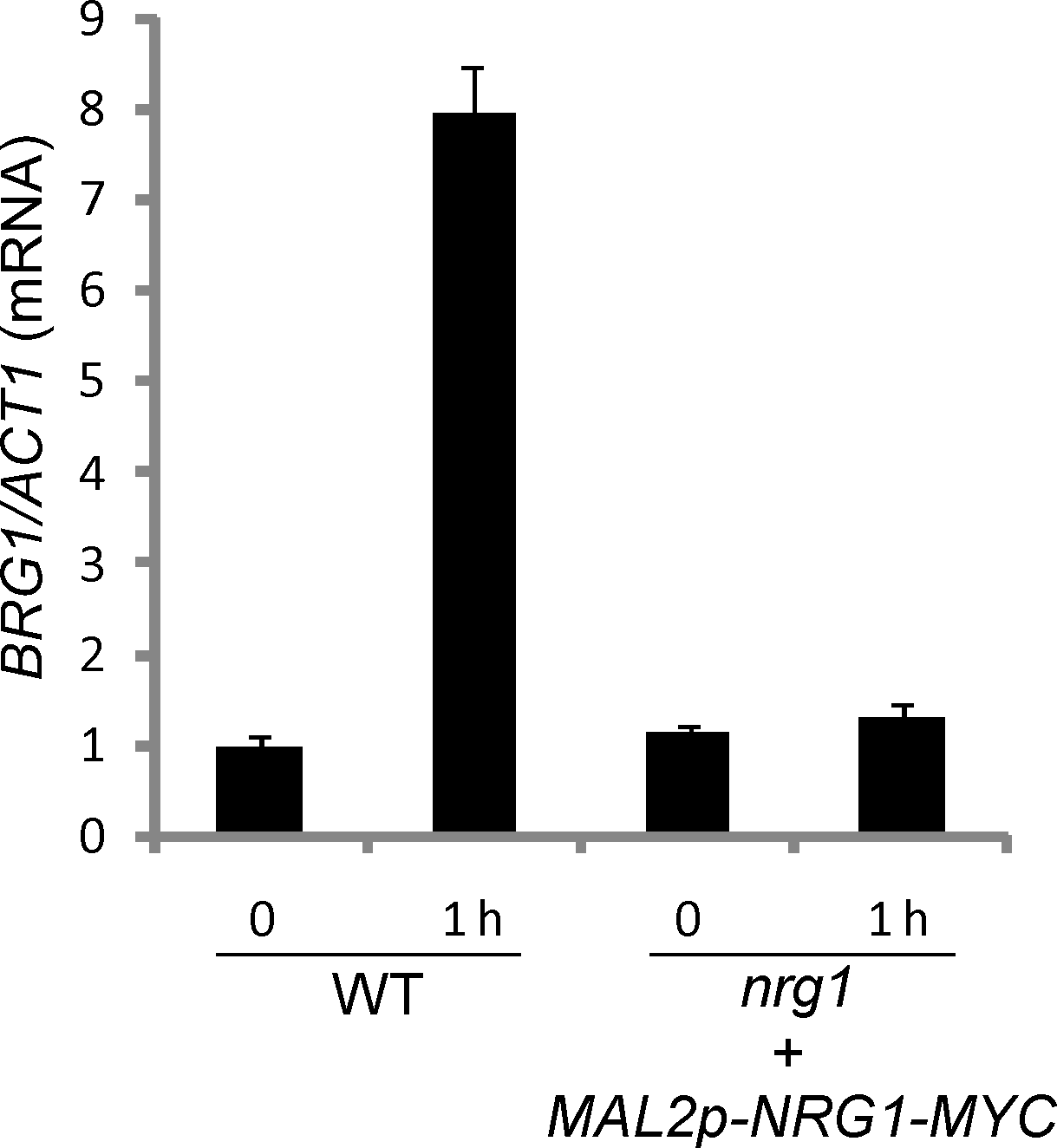

Supplement: Figure S5 — Constitutively expressed NRG1 blocks BRG1 activation. Cells of wild type or the nrg1 mutant carrying MAL2p-Nrg1-Myc, from overnight cultures in YEP Maltose at 30°C, were inoculated at 1∶20 dilution into YEP Maltose at 37°C in the presence of 10 nM rapamycin and grown for 1 h. BRG1 mRNA levels were determined by RT-PCR as Fig. 3A. (TIF) [file ppat.1002663.s005.tif]

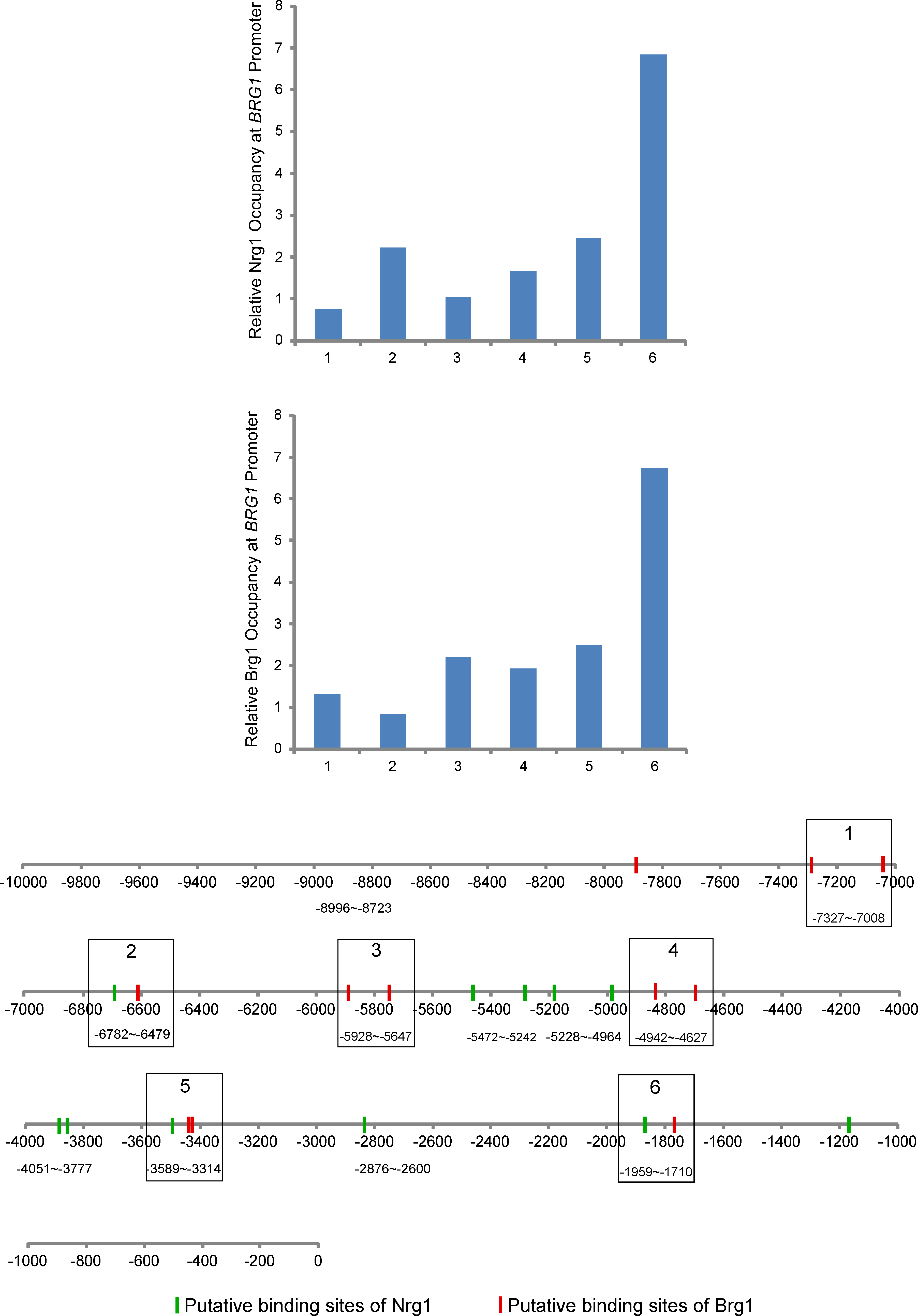

Supplement: Figure S6 — ChIP of Nrg1 and Brg1 at different regions of the BRG1 promoter. Wild-type cells carrying Nrg1-Myc or Brg1-Myc were grown in YPD medium at 25°C or YPD+10 nM rapamycin at 37°C, respectively, for 6 h. SC5314 was used as a no tag control. The enrichment over that of untagged controls is shown. Locations of specific sequence elements are marked (Nrg1 sites [green]: [A/C][A/C/G]CCCT, CCCCT, or CCCTC [20]. Brg1 sites [red]: [A/C]GGTA[C/A] [46]. Positions of primer pairs are indicated in rectangles. (TIF) [file ppat.1002663.s006.tif]

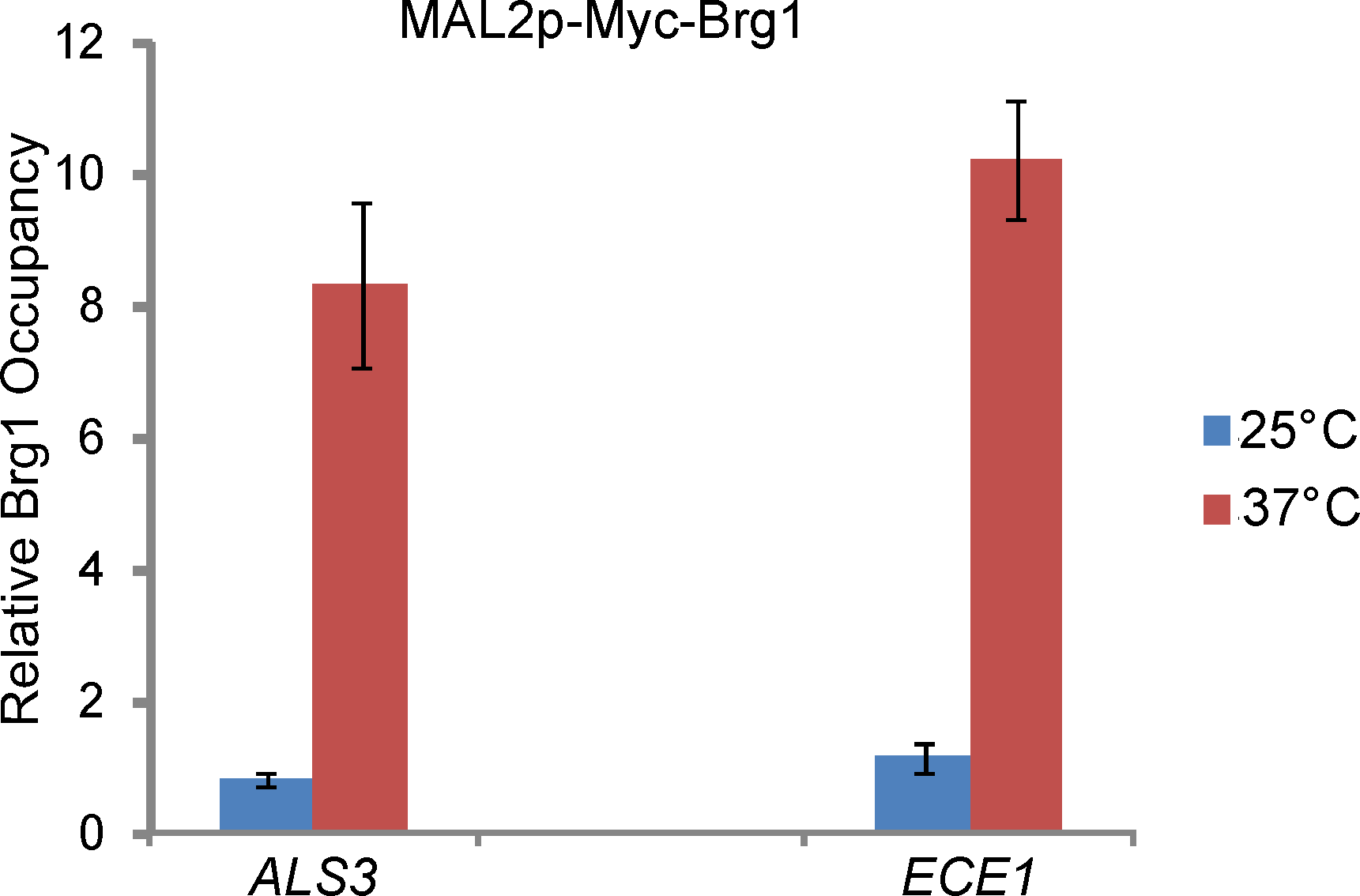

Supplement: Figure S7 — Ectopically expressed Brg1 could not bind to hyphal promoters in yeast cells. ChIP of Myc-Brg1 under the MAL2 promoter in wild-type cells grown in YEPMaltose medium at 25°C or 37°C for 6 h. (TIF) [file ppat.1002663.s007.tif]

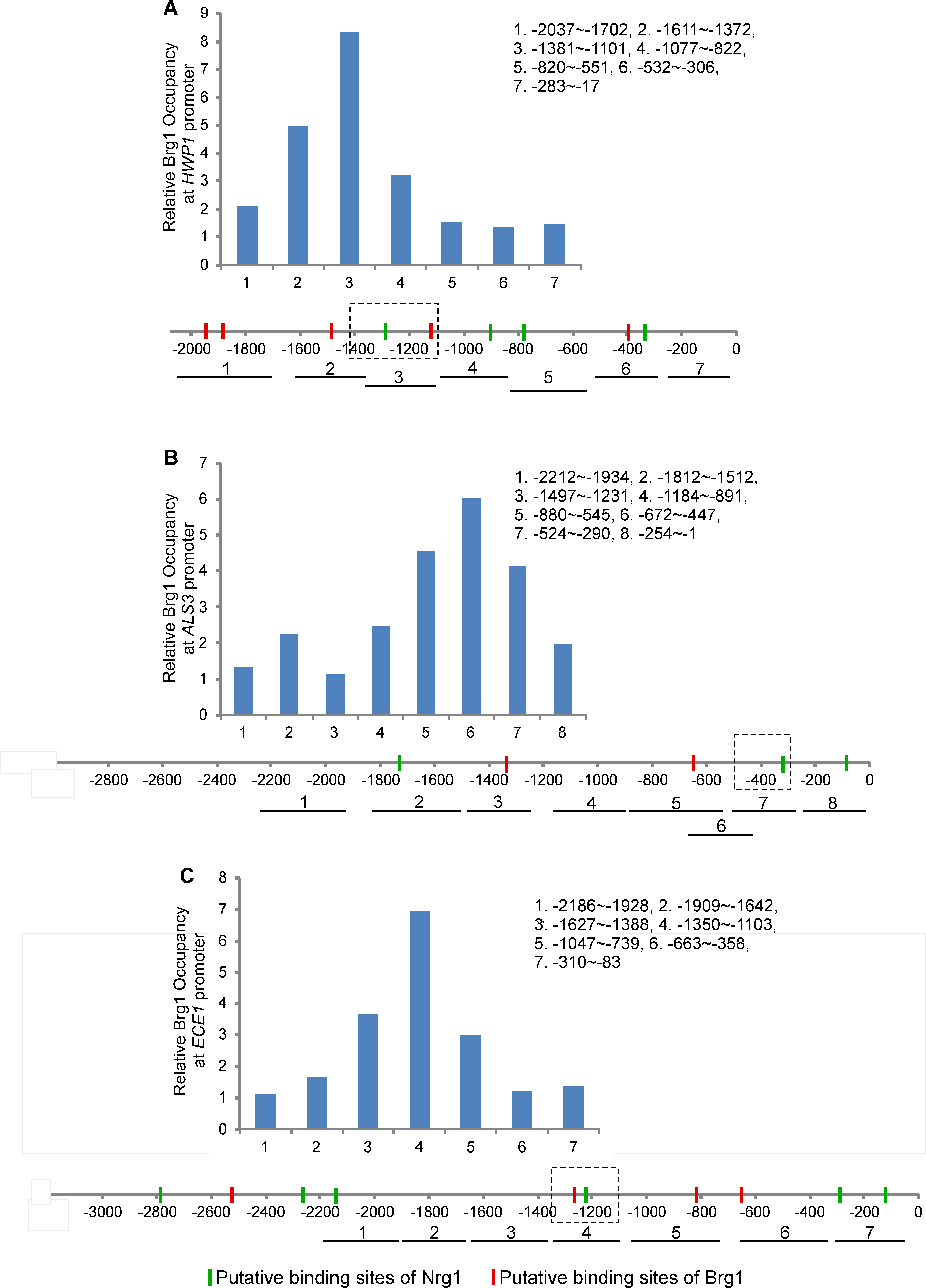

Supplement: Figure S8 — ChIP of Brg1 at different regions of hyphal promoters. Wild-type cells carrying Brg1-Myc were grown in YPD+10 nM rapamycin at 37°C for 6 h. SC5314 was used as a no tag control. The enrichment over that of untagged controls is shown. Locations of specific sequence elements are marked (Nrg1 sites [green]: [A/C][A/C/G]CCCT, CCCCT, or CCCTC [20]. Brg1 sites [red]: [A/C]GGTA[C/A]) [46]. Positions of primer pairs used for qPCR are indicated and numbered. The UAS region of each promoter is located in the dashed rectangle. (TIF) [file ppat.1002663.s008.tif]

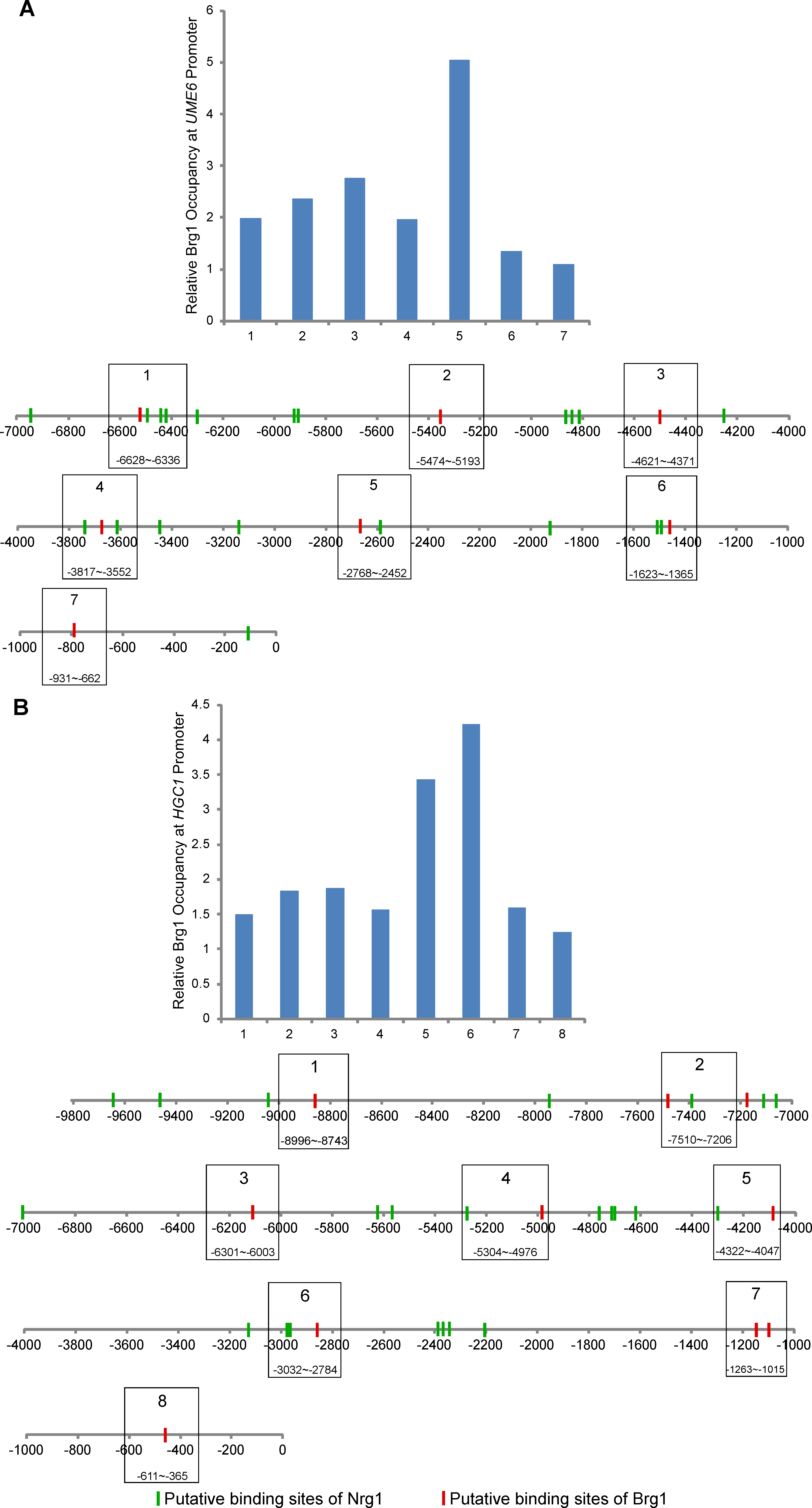

Supplement: Figure S9 — ChIP of Brg1 at different regions of UME6 and HGC1 promoters. Wild-type cells carrying Brg1-Myc were grown in YPD+10 nM rapamycin at 37°C for 6 h. SC5314 was used as a no tag control. Locations of specific sequence elements are marked (Nrg1 sites [green]: [A/C][A/C/G]CCCT, CCCCT, or CCCTC [20]. Gat2 sites [red]: [A/C]GGTA[C/A]) [46]. Positions of primer pairs used for qPCR are indicated and numbered. The enrichment of Brg1 at the indicated regions upstream of UME6 (A) and HGC1 (B) over that of untagged control is shown. (TIF) [file ppat.1002663.s009.tif]
