# Supplementary material for: A GATA Transcription Factor Recruits Hda1 in Response to Reduced Tor1 Signaling to Establish a Hyphal Chromatin State in Candida albicans
Source: PLoS Pathog. 2012 Apr 19;8(4):e1002663. doi: 10.1371/journal.ppat.1002663 (PMC3334898; doi:10.1371/journal.ppat.1002663)
Supplement: Table S1 — C. albicans strains used in this study. (DOC) [file ppat.1002663.s010.doc]

| Strain | Parent / background | Genotype | Source |
| --- | --- | --- | --- |
| SC5314 |  | Wild type | [1] |
| CAI4 | SC5314 | *ura3::1 imm434/ura3::1 imm434* | [1] |
| SN148 | SC5314 | *arg4*/*arg4* *leu2*/*leu2* *his1*/*his1*  *ura3::1 imm434/ura3::1 imm434* | [2] |
| SN250 | SC5314 | *arg4*/*arg4* *leu2::HIS1*/*leu2* *:: LEU2 his1*/*his1*  *ura3::1 imm434/ura3::1 imm434* | [3] |
| HLY3636 | SN148 | *arg4*/*arg4* *leu2*/*leu2* *his1*/*his1*  *ura3::1 imm434/ura3::1 imm434 ADE2/ade2*::*MAL2p-MYC-BRG1-URA3* | This study |
| HLY4081 | HLY3636 | *arg4*/*arg4* *leu2*/*leu2* *his1*/*his1*  *ura3::1 imm434/ura3::1 imm434 ADE2/ade2*::*MAL2p-13MYC-BRG1-URA3 HDA1/HDA1-TAP-HIS1* | This study |
| HLY3922 | CAI4 | *ura3::1 imm434/ura3::1 imm434 NRG1/NRG1-13MYC-URA3* | [4] |
| HLY4082 | CAI4 | *ura3::1 imm434/ura3::1 imm434 BRG1/BRG1-13MYC-URA3* | This study |
| MMC4 | CAI4 | *ura3::1 imm434/ura3::1 imm434 nrg1::hisG/nrg1::hisG* | [5] |
| HLY 4031 | MMC4 | *ura3*::*1 imm434/ura3*::*1 imm434 nrg1::hisG/nrg1::hisG*  *ADE2/ade2*::*MAL2p-NRG1-13MYC-URA3* | [4] |
| HLY4080 | MMC4 | *ura3*::*1 imm434/ura3*::*1 imm434 nrg1::hisG/nrg1::hisG*  *ADE2/ade2*::*MAL2p-13MYC-BRG1-URA3* | This study |
| HLY4032 | HDho15 | *MTL α/α ade2/ade2*, *ura3*::*ADE2*/*ura3*::*ADE2*, *hda1*::CAT/*hda1*::hisG | [4] |
| HLY4033 | CAI4 | *ura3::1 imm434/ura3::1 imm434 HDA1/HDA1-13MYC-URA3* | [4] |
| UZ43 | UZ24 | leu2/leu2 his1/his1 URA3/ura3  ume6*::*HIS1/ume6*::*LEU2 | [6] |
| HLY4076 | HLY4032 | *MTL α/α ade2/ade2*, *ura3*::*ADE2*/*ura3*::*ADE2*, *hda1*::CAT/*hda1*::hisG  *ADE2/ade2*::*MAL2p-UME6-13MYC-URA3* | This study |
| HLY4077 | HLY4032 | *MTL α/α ade2/ade2*, *ura3*::*ADE2*/*ura3*::*ADE2*, *hda1*::CAT/*hda1*::hisG  *ADE2/ade2*::*MAL2p-BRG1-13MYC-URA3* | This study |
| HLY4078 | CAI4 | *ura3::1 imm434/ura3::1 imm434 UME6/UME6-13MYC-URA3* | This study |
| HLY4079 | SN148 | *arg4*/*arg4* *leu2*/*leu2* *his1*/*his1*  *ura3::1 imm434/ura3::1 imm434*  *BRG1/BRG1-13MYC-HIS1*  *HWP1/HWP1p mutant-GFP-URA3* | This study |

Table S1. *C. albicans* strains used in this study

1 Fonzi, W. A. and Irwin, M. Y. (1993) Isogenic strain construction and gene mapping in Candida albicans. Genetics **134**, 717-728

2 Noble, S. M. and Johnson, A. D. (2005) Strains and strategies for large-scale gene deletion studies of the diploid human fungal pathogen Candida albicans. Eukaryot Cell **4**, 298-309

3 Noble, S. M., French, S., Kohn, L. A., Chen, V. and Johnson, A. D. Systematic screens of a Candida albicans homozygous deletion library decouple morphogenetic switching and pathogenicity. Nat Genet **42**, 590-598

4 Lu, Y., Su, C., Wang, A. and Liu, H. Hyphal development in Candida albicans requires two temporally linked changes in promoter chromatin for initiation and maintenance. PLoS Biol **9**, e1001105

5 Murad, A. M., Leng, P., Straffon, M., Wishart, J., Macaskill, S., MacCallum, D., Schnell, N., Talibi, D., Marechal, D., Tekaia, F., d'Enfert, C., Gaillardin, C., Odds, F. C. and Brown, A. J. (2001) NRG1 represses yeast-hypha morphogenesis and hypha-specific gene expression in Candida albicans. Embo J **20**, 4742-4752

6 Zeidler, U., Lettner, T., Lassnig, C., Muller, M., Lajko, R., Hintner, H., Breitenbach, M. and Bito, A. (2009) UME6 is a crucial downstream target of other transcriptional regulators of true hyphal development in Candida albicans. FEMS Yeast Res **9**, 126-142
