# Supplementary material for: A GATA Transcription Factor Recruits Hda1 in Response to Reduced Tor1 Signaling to Establish a Hyphal Chromatin State in Candida albicans
Source: PLoS Pathog. 2012 Apr 19;8(4):e1002663. doi: 10.1371/journal.ppat.1002663 (PMC3334898; doi:10.1371/journal.ppat.1002663)
Supplement: Table S2 — Primers used in this study. (DOC) [file ppat.1002663.s011.doc]

| Primer | Sequence | Purpose and feature |
| --- | --- | --- |
| 1 | 5’-CGGGATCCCACAACCAGTGCACGTATTCG | pPR673-BRG1 |
| 2 | 5’-GGCGACGCGTCGACATATGGTTGTTTGTTGCT |  |
| 3 | 5’- CGACGCGTTCCAGTTCATCATCTTTATCTTC | pMAL2-MYC-BRG1 |
| 4 | 5’CGGGGTACC**TTAATGACGAATTAAAGGAATTTGG** |  |
| 5 | 5’-CGAGATCTCCATGTAACACCAGCCGGATA | pHDA1-HDA1-TAP |
| 6 | 5’-GGCGACGCGTCGATCTTCGGAAGAGGAGTAGT |  |
| 7 | 5’-CTG TCTAGA ATGATTACCCATATGGTTAC | pMAL2-UME6-MYC |
| 8 | 5’-CTG ACGCGT ATCATTGGTTATATCATTACT |  |
| 9 | 5’- AGCTGGTGTGCCACCTCCAC | *BRG1*-qRTPCR |
| 10 | 5’- TACCACACCTGTGACATCTG |  |
| 11 | 5’-TGGTGATGGTGTTACTCACG | *ACT1*-qRTPCR |
| 12 | 5’-GACAATTTCTCTTTCAGCAC |  |
| 13 | 5’-TCTTACCTCAATCAGCATTA | *UME6-*qRTPCR |
| 14 | 5’-CAGCACTAACACTGACACC |  |
| 15 | 5’-CGGGATCCCGGATTGGTAAAGCAACAACA | pPR673-UME6 |
| 16 | 5’-GGCGACGCGTCGATCATTGGTTATATCATTAC |  |
| 17 | 5’- GTCGGTACCTTATCGGGTGATTAATAACA | pHWP1*mutant*-GFP |
| 18 | 5’- AACTCGGCTAGTTTCATTTCCTTTTTTTTTCAATTGTTCA |  |
| 19 | 5’- GAAATGAAACTAGCCGAGTTGCCTAACCATTGAAAATAAT |  |
| 20 | 5’- GTCATCGATATTGACGAAACTAAAAGCGAG |  |
| 21 | 5’- CACATAAATTGCGGATAAAC | pHWP1*mutant*-ChIP |
| 22 | 5’- TGGTTAGGCAACTCTTACCT |  |
| 23 | 5’- TGGTTAGGCAACTCGGCTAG |  |

Table S2. Primers used in this study

Restriction sites are underlined.
